# What do we know about the microbiome of *I. ricinus*?

**DOI:** 10.3389/fcimb.2022.990889

**Published:** 2022-11-16

**Authors:** Richard Hodosi, Maria Kazimirova, Katarina Soltys

**Affiliations:** ^1^ Department of Microbiology and Virology, Faculty of Natural Sciences, Comenius University in Bratislava, Bratislava, Slovakia; ^2^ Institute of Zoology, Slovak Academy of Sciences, Bratislava, Slovakia; ^3^ Comenius University Science Park, Comenius University in Bratislava, Bratislava, Slovakia

**Keywords:** *I. ricinus*, microbiome, symbiont, pathogen, interactions, vitamin B

## Abstract

*I. ricinus* is an obligate hematophagous parasitic arthropod that is responsible for the transmission of a wide range of zoonotic pathogens including spirochetes of the genus *Borrelia, Rickettsia* spp., *C. burnetii*, *Anaplasma phagocytophilum* and *Francisella tularensis*, which are part the tick´s microbiome. Most of the studies focus on “pathogens” and only very few elucidate the role of “non-pathogenic” symbiotic microorganisms in *I. ricinus*. While most of the members of the microbiome are leading an intracellular lifestyle, they are able to complement tick´s nutrition and stress response having a great impact on tick´s survival and transmission of pathogens. The composition of the tick´s microbiome is not consistent and can be tied to the environment, tick species, developmental stage, or specific organ or tissue. Ovarian tissue harbors a stable microbiome consisting mainly but not exclusively of endosymbiotic bacteria, while the microbiome of the digestive system is rather unstable, and together with salivary glands, is mostly comprised of pathogens. The most prevalent endosymbionts found in ticks are *Rickettsia* spp., *Ricketsiella* spp., *Coxiella*-like and *Francisella*-like endosymbionts, *Spiroplasma* spp. and *Candidatus* Midichloria spp. Since microorganisms can modify ticks’ behavior, such as mobility, feeding or saliva production, which results in increased survival rates, we aimed to elucidate the potential, tight relationship, and interaction between bacteria of the *I. ricinus* microbiome. Here we show that endosymbionts including *Coxiella*-like spp., can provide *I. ricinus* with different types of vitamin B (B2, B6, B7, B9) essential for eukaryotic organisms. Furthermore, we hypothesize that survival of *Wolbachia* spp., or the bacterial pathogen *A. phagocytophilum* can be supported by the tick itself since coinfection with symbiotic *Spiroplasma ixodetis* provides *I. ricinus* with complete metabolic pathway of folate biosynthesis necessary for DNA synthesis and cell division. Manipulation of tick´s endosymbiotic microbiome could present a perspective way of *I. ricinus* control and regulation of spread of emerging bacterial pathogens.

## 1 Introduction

Ticks are ubiquitous arthropod species belonging to the Ixodida order. They are hematophagous obligate ectoparasites and can be found in a high variety of habitats, ranging from driest (Foughali et al., 2021; Abdulsalam et al., 2022) to most humid (Kim et al., 2018; Yang et al., 2020; Dantas-Torres et al., 2021). Ixodida are represented by two major families: Argasidae (soft ticks) and Ixodidae (hard ticks) (Guglielmone et al., 2010). Majority of tick species belong to the Ixodidae family (Zachary, 2017). Their life cycle consists of four developmental stages (egg, larva, nymph, and adult) (Leal et al., 2020), and each active stage requires a single blood meal.

Due to their relatively low mobility, most ixodid ticks including *Ixodes* spp. adopt the questing strategy, where they climb on top of vegetation waiting for a suitable host (Nicholson et al., 2019). Ixodid ticks have extended feeding times, lasting from hours to weeks, depending on the species and developmental stage (Richter et al., 2013; Sonenshine and Anderson, 2014; Vancová et al., 2020). They have developed different feeding strategies that depend on the habitat and/or the opportunity of contact with the appropriate host. Based on the number of hosts that ticks feed on during their parasitic life cycle, they are classified into one-, two- and three-host ticks (Taylor and Coop, 2015). *I. ricinus*, the most common species in Europe, is a three host tick having a wide range of hosts with over 300 terrestrial vertebrate species (Gray et al., 2021). The generation time of *I. ricinus* is on average three years, albeit it can vary from two to six years (Anderson and Magnarelli, 2008).

Internal organs of a tick are placed in an open cavity and are surrounded by hemolymph which consists of plasma and hemocytes. Hemolymph circulation is aided by a simple “heart”. The main internal organs of ticks comprise the digestive tract, salivary glands, reproduction organs, Malpighian tubules, respiratory system and central nervous system (CNS). The CNS is fused into a single compact organ, synganglion, located in the anterior ventral region of the body (Sonenshine and Anderson, 2014). Paired salivary glands contain large grape-like clusters, known as acini, located in the anterolateral area of the body cavity. During feeding, salivary glands significantly expand in size (up to 25 times) and act as a complex multifunctional organ that regulates water balance and production of saliva and “cement”. Cement is used for the attachment of ticks to the host skin. Salivary glands products are injected *via* the tick mouthparts into the host skin. This route is pivotal for transmission of majority of tick-borne pathogens (Bowman et al., 2008; Sonenshine and Anderson, 2014). Given the high complexity of tick saliva, it carries many functions, including anti-haemostatic, anti-inflammatory, anti-wound healing, immunomodulatory and vasoactive (Prevot et al., 2006; Oliveira et al., 2011a; Pekáriková et al., 2015; Scholl et al., 2016; Tirloni et al., 2019; Zhou et al., 2020; Aounallah et al., 2021). The tick alimentary system is divided into three primary regions: the preoral canal and foregut, the midgut, and the hindgut. The midgut is the largest organ in the body of feeding ticks. The segmented midgut is well adapted to accommodate an enormous amount of host blood and fills most of the internal body space. Since digestion in ticks occurs intracellularly, the midgut also functions as a storage organ, enabling continuous digestion of its content over long periods (Sonenshine and Anderson, 2014; Mahmood et al., 2020). The hindgut is composed of the intestine, the rectal sac, a large bulbous excretory organ, and the rectum (Sonenshine and Anderson, 2014; Šimo and Park, 2014). The female reproductive system consists of U-shaped ovaries located in the posterior region of the body, paired oviducts, uterus, and vagina. Ovaries in unfed females appear as a thin band of cells and in fed females as a large organ with multiple oocytes of differing sizes (Sonenshine and Anderson, 2014).

As each animal species, ticks also possess specific microbial communities - microbiomes. A microbiome is defined as a community of commensal, symbiotic, and pathogenic microorganisms that inhabit assorted niches in the host’s body (Hooper and Gordon, 2001). In addition to eubacteria, the microbiome can be also composed of *Archaea*, viruses, and eukaryotic microorganisms such as protozoa or fungi. Transmission of maternal microbiota to the offspring, proved in humans (Dunn et al., 2017) and mice (Jašarević et al., 2021), constitutes a foundation for a healthy microbiome. In ticks, besides transovarially transmitted endosymbiotic bacteria (Baldridge et al., 2009), the maternal microbiome may provide initial inoculum of other microorganisms in eggs and developing larvae (Narasimhan et al., 2014).

Despite a diet limited to the host blood, the tick microbiome appears to be quite complex (Carpi et al., 2011; Pollet et al., 2020; Narasimhan et al., 2021; Rousseau et al., 2021; Kumar et al., 2022a). Tick microbial communities were found to include tick-borne pathogens causing diseases in humans and animals (viruses, bacteria, protozoa) or pathogens and parasites that infect ticks (microsporidia, fungi, nematodes, hymenopteran parasitoids), and non-pathogenic microorganisms such as commensals or endosymbiotic, mutualistic microbes comprising mainly bacteria. The diversity of the tick microbiome can be tied to tick species, its life stage, sex, and specific organ or tissue (Andreotti et al., 2011; Menchaca et al., 2013; Budachetri et al., 2014; Van Treuren et al., 2015; Zolnik et al., 2016; Pollet et al., 2020; Batool et al., 2021; Krawczyk et al., 2022). It may also be dependent on the geographical region, environment (soil and plants), season, and since ticks are obligate hematophagous ectoparasites, also on the microbiota of the host’s skin and blood (Carpi et al., 2011; Lalzar et al., 2012; Van Treuren et al., 2015; Gurfield et al., 2017; Swei and Kwan, 2017; Krawczyk et al., 2022).

Introducing high-throughput “omics” approaches can be considered as a milestone not only in analyzes of tick genomes and proteomes, but also of tick microbiomes (Valenzuela, 2002; Carpi et al., 2011; Michelet et al., 2014; Greay et al., 2018). Although in this field significant progress has been made, there is still a big knowledge gap regarding the tissue-specific microbiota identification, microbiota member characterization and functional role detection. *In vitro* studies and/or single-cell analysis that could help to fill in the gap in the understanding of the intra microbiota community interaction as well as inter host (tick) – microbiota interactions are still challenging.

## 2 Tick microbial coinhabitants

Beneficial symbiotic organisms can be separated into two categories: primary and secondary symbionts. Primary, or obligate, symbionts, mutualists, are essential organisms and are ubiquitous throughout the host population. Many obligate symbionts of ticks are intracellular endosymbionts (Duron et al., 2018; Ben-Yosef et al., 2020; Foughali et al., 2021), while secondary symbionts are usually extracellular (Guizzo et al., 2022). Secondary, or facultative, symbiotic organisms have the ability to improve host fitness or ecological traits but are not vital for the host’s survival. Facultative symbionts often lack specialization and various microorganisms can fulfil their secondary role (Salcedo-Porras et al., 2020). In symbiotic relations a genomic complementarity of biochemical pathways, which is required for the survival of both host and symbiotic organisms is often found (Hansen and Moran, 2011). Bacterial endosymbionts can be found in different host organs inside host cells (Klyachko et al., 2007; Qiu et al., 2014; Guizzo et al., 2017; Al-Khafaji et al., 2020; Oliver et al., 2021).

**Figure 1:**
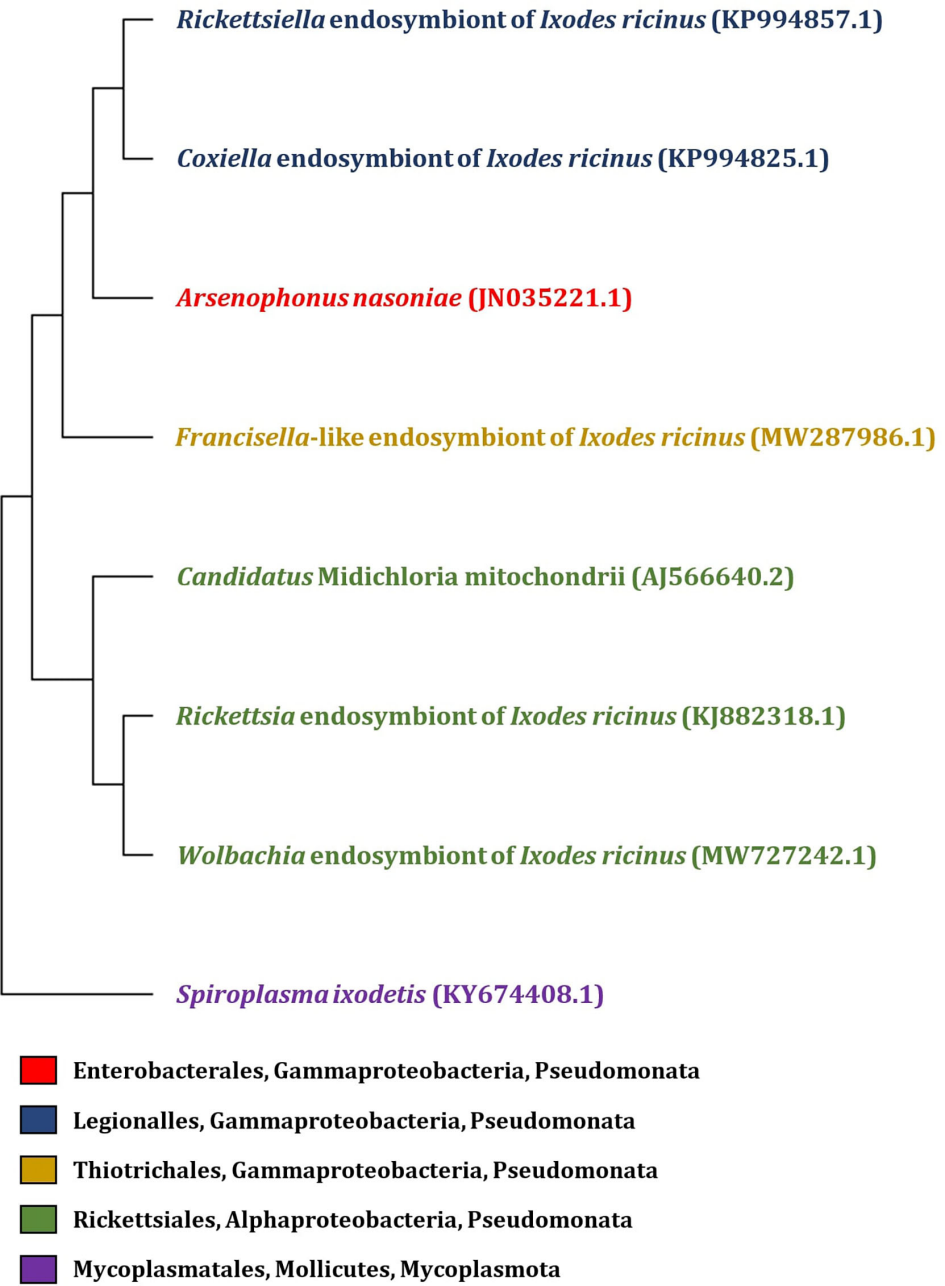
Phylogenetic tree of endosymbiotic bacteria based on *16S rRNA* gene identified in *I. ricinus*. The phylogenetic tree was constructed by MEGA 11 program, with partial *16S rRNA* gene sequence of endosymbionts isolated from the tick *I. ricinus* (the GenBank accession numbers are listed in the figure). Sequences were aligned using Muscle (Multiple Sequence Alignement) and the tree was constructed by Neighbor-joining method with Maximum Composite Likelyhood model.

In addition to tick-borne pathogens, a diverse group of commensal and symbiotic microorganisms is present within the tick microbiome (Carpi et al., 2011; Williams-Newkirk et al., 2014; Duron et al., 2017). Since the majority of tick microbiome research has been aimed towards pathogens, the biology of symbionts and their effects on ticks remain largely unexplored. However, non-pathogenic microorganisms may play a role in nutritional adaptation, reproduction, development, immunity or aiding the transmission of tick-borne pathogens (Bonnet et al., 2017). Among the tick symbionts, *Coxiella*-like endosymbionts, *Rickettsia*-like endosymbionts (Noda et al., 1997), *Francisella*-like endosymbionts (Sun et al., 2000), *Cand.* Midichloria mitochondrii (Sassera et al., 2008), *Arsenophonus*-like endosymbionts (Clay et al., 2008; Dergousoff and Chilton, 2010), *Rickettsiella* spp. (Garcia-Vozmediano et al., 2022), and *Wolbachia* spp. (Zhang et al., 2011) are included (**Figure 1**).

### 2.1 *Coxiella*-like endosymbionts

Genus *Coxiella* contains only a single validly described species, *C. burnetii* (Shaw and Voth, 2019). *C. burnetii* is an obligate intracellular bacterium (Maurin and Raoult, 1999) and the etiological agent of Q fever in humans and animals. Q fever is a worldwide distributed zoonotic disease, with symptoms including fever, hepatitis and respiratory complications (Raoult, 1993). Although, many nonpathogenic Legionellales evolved together with the nonvertebrate hosts, ticks play an important role in the circulation of *C. burnetii* in natural foci and are partly responsible for the dissemination of the infection among animals (Psaroulaki et al., 2014; Duron et al., 2018; Špitalská et al., 2018). *Coxiella*-like bacteria have a certain degree of identity with *C. burnetii*, but this identity is not sufficient to consider them as the same species (Angelakis et al., 2016). Based on the sequence of the small ribosomal subunit (*16S rRNA*) gene, all *Coxiella*-like bacteria belong to the *Coxiella* genus (Gammaproteobacteria). The identity between *C. burnetii* and *Coxiella*-like bacteria was found to range from 91% to 98% (Zhong, 2012). Whole genome sequencing and multi-locus typing analyses showed that *Coxiella*-like bacteria represent a monophyletic and ancient group allied to ticks. *Coxiella burnetti* originated from progenitors of endosymbiotic *Coxiella* sp. hosted by ticks and was derived from a rare and quite recent event, likely by acquisition of virulence factor (Duron et al., 2015). Probably because of different approaches for *C. burnetii* detection, according to available literature, out of several studies on *I. ricinus*, only in one *C. burnetii* was detected.

Tick *Coxiella*-like endosymbionts (*Coxiella-*LE) cannot be found associated with any specific tissue. They are located across the majority of tick organs, however, organs that are infected most likely and in the highest concentration are Malpighian tubules and ovaries. Significant distribution of *Coxiella*-LE in tick reproductive organs suggests they are primarily transmitted transovarially and also transtadially (Clay et al., 2008; Zhong, 2012). Vertically transmitted *Coxiella-*LE that were detected in eggs and larvae of different individuals, beared 98% identity of the *16S rRNA* sequence. Similar to other endosymbionts, localization of *Coxiella* in ovaries may be advantageous for its survival and transmission (Almeida et al., 2012; Lalzar et al., 2014; Duron et al., 2015). High concentration of *Coxiella* in Malpighian tubules may play a role in tick nutrition. Since Malpighian tubules are predominantly involved in osmoregulation and excretion (Sonnenshine and Anderson, 2014), there is a possibility that *Coxiella-*LE are able to utilize ticks’ nitrogenous metabolites to synthesize compounds such as B vitamins (Buysse et al., 2019). Since vitamin B is essential for cell growth and development and it is involved also in energetic metabolism, we can suppose that there are higher energetic requirements of ovaries and Malpigian tubules than of other organs.

This may be connected to the high frequency of *Coxiella*-LE, e.g. in the population of *Rhipicephalus microplus* ticks (Guizzo et al., 2017). However, also a common vertebrate host may serve as *Coxiella*-LE reservoir for further infection of ticks including *Dermacentor* and *Hyalomma* species. Furthermore, almost each tick genus, e. g. *Amblyomma*, *Haemaphysalis*, and also *Ixodes* spp. including *I. ricinus* were found to be positive for *Coxiella*-LE (Guizzo et al., 2017; Rahal et al., 2020). However, microbiome analyses revealed that prevalence of the *Coxiella*-LE is quite low in *I. ricinus* averaging around 5.5%, and depending on geography, tick stage and sex. *Coxiella*-LE were detected in 12% of adult females, 4.2% adult males and 18.3% nymphs. This lower prevalence, compared to other tick species that inhabit same locations (such as *Dermacentor reticulatus* and *Heamaphysalis inermis*, whose prevalence ranges from 25 to over 50%) (Papa et al., 2017; Špitalská et al., 2018) can be explained by significantly higher prevalence of *Candidatus* Midichloria mitochondrii in *I. ricinus* (Sassera et al., 2006). This endosymbiont most likely plays similar nutritional role and is able to outgrow population of either *Coxiella*-LE or *Francisella*-LE.

Since tick’s diet consists mainly of blood and vertebrate blood rarely contains sufficient quantities of amino acids and B-vitamin, it needs to be complemented with bacterially synthesized vitamins (Zhong, 2012). Vitamin B is required for blood digestion and is essential for the survival and reproduction of ticks. *Coxiella*-LE are thought to supply missing vitamin B (Buysse et al., 2019). Biosynthetic pathways for the vitamins Biotin (B7), riboflavin (B2), Pyridoxine (B6), Folic acid (B9) and Pantothenate (B5) were found in all *Coxiella*-like bacteria (Gottlieb et al., 2015; Guizzo et al., 2017).

### 2.2 *Francisella*-like endosymbionts

*Francisella* is a facultative intracellular Gram-negative Gammaproteobacterium. Within this genus, four valid species, *Francisella tularensis*, *F. philomiragia*, *F. noatunensis*, and *F. hispaniensis*, have been recognized (Liu et al., 2016). *Francisella tularensis* is naturally found in vertebrates, invertebrates, contaminated soil, water and vegetation (Mörner, 1992) and causes a zoonotic disease called tularemia (Hightower et al., 2014). Tick vectors of *F. tularensis* include species of genera *Amblyomma*, *Dermacentor*, *Haemaphysalis*, *Ixodes* and *Ornithodoros* (Gordon et al., 1983).

Multiple tick species were found to host bacteria closely related to *F. tularensis*, called *Francisella*-like endosymbionts (*Francisella*-LE) (Dergousoff and Chilton, 2012) that have a worldwide distribution in ixodid ticks, specifically in the genera *Ixodes*, *Dermacentor* and *Amblyomma* (de Carvalho et al., 2011). Špitalská et al. (2018) identified *Francisella*-LE in *I. ricinus* from Slovakia what correlated with previous findings of Hornok et al. (2013). *Francisella*-LE are predominantly localized in Malpighian tubules in clusters, surrounding nuclei. *Francisella*-LE were also observed in the poles of oocytes and scattered in salivary gland acini, where they appear to surround nuclei (Azagi et al., 2017). *Francisella-*LE replicate intracellularly and were found in 95 to 100% of eggs of *Hyalomma* ticks, which confirmed transovarial transmission. The presence of *Francisella-*LE in salivary glands might suggest a potential nutritional role in the supply of B vitamins that are deficient in the tick’s diet. *Francisella*-LE were detected in half of the analyzed questing *I. ricinus* ticks for the first time in Slovakia in 2017 (Azagi et al., 2017; Špitalská et al., 2018) and in *I. ricinus* larvae from Hungary (Hornok et al., 2013). On average, only 2.3% of analysed individuals of *I. ricinus* carried *Francisella*-LE. The prevalence was dependent on sex and stage, with higher being in female adult ticks (6.1%) (Hornok et al., 2013; Wójcik-Fatla et al., 2015; Špitalská et al., 2018). Similar to *Coxiella*-LE, this low prevalence of *Francisella*-LE in *I. ricinus* has most likely the same explanation as mentioned above in section 2.1. So far, there is a lack of knowledge concerning the pathogenic potential of *Francisella-*LE (Špitalská et al., 2018).

Previously, a microorganism, originally described as *Wolbachia*-like symbiont, was found in nearly all examined *Dermacentor andersoni* ticks, and based on the 16S rRNA sequence, it was characterized as an endosymbiont belonging to the genus *Francisella* with 95.4% identity to pathogenic *F. tularensis* (Niebylski et al., 1997a).

### 2.3 *Rickettsia* endosymbionts

*Rickettsia* spp. are Gram-negative obligate intracellular bacteria belonging to the class Alphaproteobacteria and include multiple known arthropod-borne human pathogens (Guillotte et al., 2021). *Rickettsia* spp. can occur both as vertebrate pathogens and arthropod symbionts, have a wide diversity and are classified into at least four groups: ancestral group (AG), typhus group (TG), transitional group (TRG), and spotted fever group (SFG) rickettsiae (Gillespie et al., 2012).

Rickettsiae of undetermined pathogenicity are commonly detected in ticks. Some are labelled as non-pathogenic beneficial endosymbionts and some are considered as potential pathogens (Kurtti et al., 2016). Analysis of the RIES (rickettsial endosymbiont of *Ixodes scapularis*) genome showed vast disruption of genome caused by mobile genetic elements and acquisition of genes responsible for intracellular parasitism. Over one-third of the REIS genome is made up of mobile genetic elements and transposases (Gillespie et al., 2012; Thorpe et al., 2021). *Rickettsia buchneri* and *Rickettsia peacockii* are non-pathogenic rickettsial endosymbionts of *I. scapularis* and *Dermacentor andersoni*, respectively, and are spread through transovarial transmission. They are mainly associated with tick ovarian tissue and both endosymbionts are non-infectious for vertebrate cells (Niebylski et al., 1997b; Kurtti et al., 2015). Pathogenic rickettsiae, transmitted by ticks to vertebrate hosts, are closely related to these endosymbionts. *Riskettsia peacockii* is closely related to *R. rickettsii* (causative agent of Rocky Mountain spotted fever), and *R. buchneri* to *R. monacensis* transmitted by *I. ricinus* (Simser et al., 2002; Kurtti et al., 2015). Compared to the related pathogens, both endosymbionts possess an extensively rearranged genome with gene mutations attenuating virulence and the capability to cause cytopathic effects (Kurtti et al., 2016). There is insufficient of research aimed towards detection of *Rickettsia* endosymbionts in *I. ricinus*. *Rickettsia* endosymbionts were identified in *I. ricinus* in Poland with prevalence of 20% (Stańczak et al., 2018), and also in Eastern Slovakia (Špitalská et al., 2020), nonetheless their interactions with ticks are unknown.

### 2.4 *Rickettsiella* spp.

*Rickettsiella* species (class Gammaproteobacteria, order Legionellales, family Coxiellaceae) are obligate intracellular bacteria that are associated with many arthropod species, including ticks. They are maternally inherited and, depending on the species and their arthropod host, they are either pathogenic with negative effects on the development and reproduction of arthropods (Rosenwald et al., 2020), or are essential for their survival, e.g. by supplying B-vitamins (Price et al., 2021). In contrast to insects and other tick species, only a few studies have dealt with presence of *Rickettsiella* in *I. ricinus*, while their biological role in this tick species is unknown (Carpi et al., 2011; Duron et al., 2015; Cerutti et al., 2018; Estrada-Peña et al., 2018; Aivelo et al., 2019; Garcia-Vozmediano et al., 2022). Recently, *I. ricinus* from Belgium, Italy, the Netherlands, Sweden, and the UK were screened for the presence of *Rickettsiella* spp. and their genetic diversity was studied (Garcia-Vozmediano et al., 2022). Moreover, associations of *Rickettsiella* spp. with the endosymbiont *Midichloria mitochondrii* and pathogenic *Borrelia burgdorferi* s.l. and *Borrelia miyamotoi* were investigated. Presence of *Rickettsiella* spp. was confirmed in majority of the studied *I. ricinus* populations across the studied regions and in all active tick stages. But, prevalence of *Rickettsiella* spp. was highest in adult ticks and differed between the sites. Four *Rickettsiella* clades were identified, with various patterns depending on the geographic locations. No associations of *Rickettsiella* spp. with the other investigated bacteria infecting *I. ricinus* were found. Based on these results, the authors suggest that *Rickettsiella* spp. are genetically and biologically diverse facultative symbionts of *I. ricinus* and environmental factors influence their presence, prevalence and geographic distribution.

### 2.5 *Candidatus* Midichloria mitochondrii

*Candidatus* Midichloria mitochondrii, formerly known as IricES1 (*I. ricinus* EndoSymbiont 1), is a Gram-negative Alphaproteobacterium, a member of the order Rickettsiales and intracellular endosymbiont of *I. ricinus* (Comandatore et al., 2021). The bacterium was detected in 100% of female ticks while only 44% of males were infected. Males, if infected, harbor a smaller quantity of bacteria than females. *Cand*. Midichloria mitochondrii was detected in every egg, suggesting that the major route of transmission is transovarial (Lo et al., 2006). Real-time PCR analysis showed the presence of *Cand*. Midichloria mitochondrii in every development stage (eggs, larvae, nymphs and adults) of *I. ricinus* (Sassera et al., 2006).

The bacterium resides in the mitochondria of tick ovaries, namely in the periplasmatic space between two membranes of these organelles. During the development of oocytes, the bacteria consume the inner part of mitochondria and reproduce inside (Sassera et al., 2006). According to the “mitochondrion-to-mitochondrion hypothesis”, the bacterium may be able to move in-between mitochondria, possibly within a mitochondrial network (Comandatore et al., 2021). The number of bacteria was observed to increase during feeding, which suggests a possible role in the metabolism of tick’s blood meal (Sassera et al., 2008). Tick populations maintained in the laboratory after a few generations eventually lost the symbiont. This can indicate that its role is important in natural habitat, e.g. for survival in a cold climate (Lo et al., 2006). Sequencing of *Cand.* Midichloria mitochondrii genome showed the presence of unique gene sets found in no other Rickettsiales. Among these was a cbb(3)-type cytochrome c oxidase, a haem-copper proton-pumping oxidase with the ability to perform oxidative phosphorylation at low oxygen tension. Due to its high oxygen affinity, this enzyme can allow respiration under microaerobic conditions, thus aiding ATP production when oxygen availability is scarce. This suggests that the bacterium could serve as a source of ATP for the tick under low-oxygen conditions (Sassera et al., 2011). *Cand.* Midichloria mitochondrii also possess complete metabolic pathways for *de novo* biosynthesis of vitamin B, which is necessary for tick development and is lacking in its blood meal (Sassera et al., 2011; Al-Khafaji et al., 2019).

In addition to *I. ricinus*, the distribution of *Cand.* Midichloria related endosymbionts was found in eight other hard tick species, though the prevalence levels differed (Epis et al., 2013).

### 2.6 *Spiroplasma* spp.

*Spiroplasma* spp. are helical bacteria and belong to the family Spiroplasmataceae, order Mycoplasmatales and the class Mollicutes (Tully et al., 1993). Bacteria of genus *Spiroplasma* are predominantly found in plants and arthropods. Some *Spiroplasma* spp. are potential pathogens of vertebrates, but most of them are symbionts (Henning et al., 2006). Only two *Spiroplasma* spp. were validated in ticks: *Spiroplasma mirum* and *Spiroplasma ixodetis* (Ogata et al., 2021). The first reported tick *Spiroplasma*, *S. mirum*, was isolated from the tick *Haemaphysalis leporispalustris* (Tully et al., 1982). *Spiroplasma ixodetis* was first found in *Ixodes pacificus* (Tully et al., 1981). The presence of a *Spiroplasma* strain, closely related to *S*. *ixodetis*, was also detected in *I. ricinus* (Tenckhoff et al., 1994; Henning et al., 2006; Bell-Sakyi et al., 2015)

### 2.7 *Arsenophonus*-like symbionts

The genus *Arsenophonus* is an endosymbiotic group of bacteria, mainly associated with insects (Nováková et al., 2009). The first isolated bacteria from this clade was *Arsenophonus nasoniae*, a male-killing symbiont of the parasitoid wasp *Nasonia vitripennis* (Gherna et al., 1991). A strain of *A. nasoniae* was also identified in ticks of genera *Dermacentor* and *Amblyomma* in the USA (Clay et al., 2008; Dergousoff and Chilton, 2012) and in nymphs of *I. ricinus* in Slovakia. Molecular screening of *I. ricinus* ticks from the same location identified *A. nasoniae* presence in 37% of nymphs and only in 3.6% of adults. The pathogenicity of this bacterium for vertebrate hosts is unknown (Clay et al., 2008; Dergousoff and Chilton, 2012; Subramanian et al., 2012). Bohacsova et al. (2016) detected *A. nasoniae* in adult *Ixodiphagus hookeri* wasps, which are parasites of *I. ricinus* nymphs. *Arsenophonus nasoniae* was not detected in tick nymphs that were not parasitized by wasps. Vertical transmission was also observed in *Arsenophonus* that was detected in eggs of *Amblyomma* ticks (Dale et al., 2006). Liu et al. (2013) detected *Arsenophonus* endosymbiont in multiple organs (ovaries, salivary glands, midgut and Malphigian tubules) of *Dermacentor reticulatus* ticks. *Arsenophonus* was present in different developmental stages (eggs, larvae, nymphs and adults), indicating possibility of vertical transmission.

### 2.8 *Wolbachia* spp.

*Wolbachia* are obligate intracellular endosymbiotic Alphaproteobacteria associated with arthropods and nematodes (Landmann, 2019). They are capable of manipulating the host reproductive system through cytoplasmic incompatibility, induction of parthenogenesis (Werren, 1997). The presence of *Wolbachia* endosymbionts in various hard ticks was reported in multiple studies (Benson et al., 2004; Andreotti et al., 2011; Thapa et al., 2019; Duan et al., 2020), however, it is uncertain if ticks themselves are infected with *Wolbachia*, or if the detected bacteria originate from parasitic wasps of the genus *Ixodiphagus* (Hu et al., 1998). The study conducted by Tijsse-Klasen et al. (2011) indicates the latter hypothesis. Presence of *I. hookeri* was detected in 9.5% of tested *I. ricinus*. *Wolbachia* was found in 87% of the ticks that were positive for *I. hookeri* and only in 1.6% of *I. hookeri*-free ticks tested positive for *Wolbachia*. Selective presence of *Wolbachia* in individual nymphs (Alafaci et al., 2021) correlates with the detection of *Wolbachia* in all of the analyzed *I. ricinus* nymphs parasitized by *I. hookeri* while all unparasitized nymphs were *Wolbachia*-free (Plantard et al., 2012). From these facts a question rose whether tick cells are capable of supporting the growth of *Wolbachia*. Khoo et al. (2020) tested *in vitro Wolbachia* infection susceptibility on cell lines derived from *I. ricinus*, *I. scapularis* and *Rhipicephalus microplus* and found that *Wolbachia* was able to invade and replicate in all tick cell lines. Zhang et al. (2011) investigated the distribution of *Wolbachia* in *Amblyomma americanum* in association witch nematodes. Association between tick parasite infection and *Wolbachia* prevalence was similar to results of Tijsse-Klasen et al. (2011). *Wolbachia* endosymbiont was significantly more prevalent in nematode-infected ticks.

Taking into consideration results from above mentioned studies, the lack of strict *Wolbachia* and tick-parasite co-infection concordance suggests that *Wolbachia* presence in ticks is not solely due to wasp or nematode infection. Furthermore, detection of *Wolbachia* endosymbiont in both female and male adult ticks and in nymphs may suggest a possible way of vertical transmission (Zhang et al., 2011; Chao et al., 2021).

Besides the reproduction system, *Wolbachia* distribution was detected also in various somatic tissues of its host, including the nervous tissue, and it might be able to regulate host behavior and physiology (Albertson et al., 2009; Albertson et al., 2013; Strunov et al., 2013).

Since it is still very difficult to identify the true microbiome it is necessary to focus also on the identification of potentially contaminating microorganisms originating in the environment, tick surface or laboratory reagents (see also **Supplementary Table 1**).

## 3 Pathogens

### 3.1 Viruses

*I. ricinus* is an important vector of viruses of public health interest (Charrel et al., 2004). Previously, research on the *I. ricinus* virome has been mainly focused on viruses from the family *Flaviviridae* that cause disease in humans, namely tick-borne encephalitis virus (TBEV), and Louping ill virus causing disease in domestic ruminants (Gritsun et al., 2003; Charrel et al., 2004; Bartíková et al., 2017). Uukuniemi phlebovirus (*Phenuiviridae*) (Papa et al., 2018), Tribeč and Lipovník virus (Kemerovo virus complex) (Grešíková, 1972) and Eyach virus (Rehse-Küpper et al., 1976) (*Reoviridae)*, associated with human diseases, and *Murid herpesvirus 4* (DNA virus, family) *Herpesviridae*) (Ficová et al., 2011) were also identified in *I. ricinus*. Recent metagenomics studies provided deeper insight into the virome composition of *I. ricinus* from several European countries and previously undetected or new viruses belonging to viral orders and families such as *Flaviviridae, Rhabdoviridae*, Bunyaviales, Mononegavirales*, Nyamiviridae, Nairoviridae, Luteoviridae, Phenuiviridae, Partiviridae, Peribunyaviridae* and *Reoviridae* were identified (Moutailler et al., 2016a; Pettersson et al., 2017; Vanmechelen et al., 2017; Kuivanen et al., 2019; Ohlendorf et al., 2019; Tomazatos et al., 2021; Sameroff et al., 2022) (see also **Supplementary Table 2**).

The knowledge on interactions between viruses and *I. ricinus* is limited (Nuttall, 2014; Kazimírová et al., 2017). The most explored and understood interaction is that between *I. ricinus* and TBEV. For example, Mansfield et al. (2017) observed induction of differential expression of genes responsible for cell survival and resistance in *I. ricinus* cells infected with TBEV and the Louping ill virus. The presence of these flaviviruses increased the gene expression of apoptosis-associated genes, and immune genes. However, an increase in expression of genes encoding proteins responsible for apoptosis inhibition was also observed, which may be influenced by pathogens to promote infection.

Tick saliva plays an important role in facilitating of TBEV transmission to the vertebrate host (i.e. salivary assisted transmission) (Nuttall and Labuda, 2008). Hart et al. (2020) found differences in the composition of the saliva between uninfected and TBEV-infected *I. ricinus* females during the early stages of feeding, particularly in the expression of uncategorized genes, proteases, Kunitz-type serine protease inhibitors, cytotoxins, and lipocalins. These changes in the tick sialome are probably significant in enhancing virus transmission to the vertebrate host. In the related species, *I. scapularis*, micro RNA expression was analyzed in salivary glands of ticks infected with Powassan virus. In virus-infected tick females, 35 salivary gland miRNAs were found to be significantly up-regulated, while 17 miRNAs were significantly down-regulated. Potential role of this difference in miRNA expression could be regulation of Powassan virus replication in host tissues (Hermance et al., 2019).

Laboratory studies also revealed that application of *I. ricinus* saliva significantly increased TBEV replication in murine dendritic cells *in vitro* and induced activation of Akt pathway (Lieskovská et al., 2018). This pathway plays an important regulatory role in numerous cellular processes and serves as anti-apoptotic signalling pathway in infected dendritic cells, which may aid TBEV replication and transmission. Another tool to explore virus-tick interactions is the study of protein-protein interactions (PPI). Lemasson et al. (2021) constructed a PPI network of TBEV and Louping ill virus, their hosts and vector, *I. ricinus*. The viral proteins interacted with numerous host cell proteins, particularly with pathways for cytoskeletal function, transcription, signal transduction and protein degradation. TBEV infection was found to change tick behavior as well in terms of higher mobility and questing activity (Alekseev et al., 1988; Belova et al., 2012).

### 3.2 Bacteria

*I. ricinus* is an important vector of a variety of infectious bacteria causing diseases in humans and domestic animals, e.g. *Borrelia burgdorferi* s. l. (Stanek et al., 2012; Strnad et al., 2017), *Borrelia miyamotoi* (Wagemakers et al., 2015)*, Anaplasma phagocytophilum* (Strle, 2004; Stuen et al., 2013) *Neoehrlichia mikurensis* (Portillo et al., 2018), *Rickettsia* spp. (Oteo and Portillo, 2012; Tomassone et al., 2018), *C. burnetii* (Rehácek et al., 1991), and *Francisella tularensis* (Hubálek and Rudolf, 2011). In most cases, these bacteria cause zoonotic diseases and humans are their incidental and dead-end hosts. They are commonly transmitted from ticks to vertebrate hosts through hematophagous bites (Hofhuis et al., 2017). There are multiple ways of bacteria propagation in tick populations - vertical (transovarial) transmission (from females to eggs and the subsequent generation) (Harris et al., 2017; Ogata et al., 2021; Tufts and Diuk-Wasser, 2021) or horizontal transmission (from tick to tick through co-feeding on a host or by feeding on an infected host) (Belli et al., 2017; Moraes-Filho et al., 2018; Nah and Wu, 2021). The vertebrate pathogens may persist for long periods in infected ticks thanks to their ability to be transmitted from one stage to another (transstadial transmission) (Kalmár et al., 2015; Răileanu et al., 2020). Co-infection with multiple pathogens of vertebrates is a common feature observed in ticks. Thus, the possibility of co-transmission of these pathogens to human or animal hosts increases. Also, tick endosymbionts may be capable to interfere with pathogen transmission (Moutailler et al., 2016b; Cutler et al., 2021). According to Špitalská et al. (2018) co-occurrence of tick endosymbionts including *Francisella*-LE and *Coxiella*-LE with pathogenic bacteria like *Rickettsia* spp. and *C. burnetii* was detected in approximately 50% of field-collected adult *D. reticulatus* ticks, but only in a few *I. ricinus* adults and nymphs. However, not only endosymbionts modulate animal pathogen colonization in ticks; also the microbiota composition of individual tick organs can lead to dysbiosis, thus the ifluence of animal pathogens and tick endosymbionts may be bidirectional (Hussain et al., 2022).

*Borrelia* are helically shaped bacteria from the phylum Spirochaetota, order Spirochaetales. They comprise the *B. burgdorferi* sensu lato (s.l.) group which includes causative agents of Lyme disease, and the group containing causative agents of tick-borne relapsing infections.

The *B. burgdorferi* s. l. complex contains currently more than 20 geno-species, and their number is still increasing. Among them, *B. burgdorferi* sensu stricto (s. s.) in the USA and *B. garinii* and *B. afzelii* in Europe and Asia are the most important agents of Lyme borreliosis in humans (Steere et al., 2016; Marques et al., 2021). The dominant vectors of *B. burgdorferi* are species of the *I. ricinus* complex - *I. ricinus* and *I. persulcatus* in Europe and Asia, and *I. scapularis* and *I. pacificus* in North America (Steere et al., 2016).

Upon the entry in the tick midgut during feeding on infected host, *B. burgdorferi* s. s. cells begin to express the Outer surface protein A (OspA), which facilitates colonization of the tick midgut (Schwan et al., 1995; Pal et al., 2000). Expression of OspA continues in the midgut of moulted unfed ticks and is essential for maintaining the bacteria in the tick body (Pal et al., 2000). OspA is required for mediating attachment of *B. burgorferi* to the tick midgut by binding to the midgut receptor called Tick Receptor for OspA (TROSPA) (Pal et al., 2004). In the course of tick feeding, the spirochetes downregulate the expression of OspA and upregulate the expression of the Outer surface protein C (OspC). OspC is a lipoprotein that facilitates migration of *B*. *burgdorferi* from the gut to salivary glands and plays also an important role in subsequent infection of the vertebrate hosts (Grimm et al., 2004). To survive initial transmission from the tick salivary glands to vertebrate hosts, *B*. *burgdorferi* s. s. requires to be coated by tick salivary protein Salp15. This protein interacts with OspC and creates a coating around bacteria. The coating with Salp15 protects *B*. *burgdorferi* from vertebrate hosts antibody mediated immunity (Ramamoorthi et al., 2005). For transmission of bacteria from the tick gut to the host, a minimum 6-hour feeding period is required (Strle et al., 1996; Stanek and Kahl, 1999).

Majority of tick-borne borreliae causing relapsing fever are transmitted by Argasidae (soft ticks) (Trevisan et al., 2021). A single causative agent of tick-borne relapsing fever in humans (also called “Borrelia miyamotoi Disease” or BMD), *Borrelia miyamotoi*, belongs to hard-tick-borne relapsing fever borreliae and is transmitted by *Ixodes* spp., particularly *I. ricinus* in Europe, *I. pacificus* and *I. scapularis* in Northern America, and *I. persulatus* in Asia (Wagemakers et al., 2017; Trevisan et al., 2021). Rodents are suggested as natural reservoirs of *B. miyamotoi.* In contrast to *B. burgdorferi* s. l., for which horizontal and transstadial transmission are known, for *B. miyamotoi* transovarial transmission was confirmed (Krause et al., 2015).

*C. burnetii* belongs to Gammaproteobacteria and is the causative agent of Q fever (see above in Section 2). As an obligate intracellular pathogen of vertebrates, the bacterium replicates inside vacuoles of eukaryotic cells (Samoilis et al., 2010; Steiner et al., 2021). *C. burnetii* is able to propagate in a great variety of invertebrate and vertebrate hosts and persist for prolonged periods outside of the host (Seshadri et al., 2003). The most common mammal reservoirs for human infections are sheep, cattle and goats. *C. burnetii* has been isolated from multiple tick species from around the world, including *I. ricinus* (Hildebrandt et al., 2011; Koka et al., 2018; González et al., 2020; Jiao et al., 2021). Even though *C. burnetii* is rarely transmitted to humans through tick bites, ticks are also considered among its vectors. These bacteria possess the ability to penetrate the tick’s digestive tract and multiply in the cells of the midgut. Ticks disperse the bacteria through feces contaminating the fur or skin of animals or transmit the bacteria through saliva (Borawski et al., 2020). Transmission to the human host is most commonly achieved through inhalation of contaminated aerosol originating from pets or farm animals.

*I. ricinus* was found to harbour a novel obligate intracellular gamma-proteobacterium, *Diplorickettsia massiliensis* (order Legionellales, family Coxiellaceae) that was isolated from questing ticks by using mammalian and amphibian cell lines (Mediannikov et al., 2010) and was found to be pathogenic to humans (Mathew et al., 2012).

Rickettsioses are caused by obligate intercellular Alphaproteobacteria belonging to the genus *Rickettsia* and are transmitted by various arthropod vectors. Genus *Rickettsia* is divided into separate groups: spotted fever group rickettsiae, typhus group rickettsiae, *Rickettsia canadensis* group and *Rickettsia belli* group (Merhej and Raoult, 2011). tick-borne rickettsioses are caused by rickettsiae of the spotted fever group (SFG) and are distributed worldwide, whereby the distribution area of a specific species coincides with that of its vector. Certain species of SFG rickettsiae are primarily associated with single tick species. The initial infection of ticks with rickettsiae occurs during feeding on infected hosts or by co-feeding with infected ticks (Philip, 1959). The reservoirs are ticks (due to transovarial and transstadial transmission) (Burgdorfer and Brinton, 1975; Podboronov and Pchelkina, 1989; Socolovschi et al., 2009a; Hosseini-Chegeni et al., 2020) and probably small mammals and birds (Elfving et al., 2010; Essbauer et al., 2018; Fischer et al., 2018). The bacteria enter tick gut cells though intracellular digestion of host blood cells. Rickettsiae are able to escape tick immune responses and invade tick hemocytes (Kurtti et al., 2005; Ceraul et al., 2007), from where they subsequently disseminate to all tissues and organs, including salivary glands (Lejal et al., 2019). Rickettsiae also invade tick oocytes (probably during active oogenesis) and ovarian tissues, what leads to transovarial (vertical) transmission (Moore et al., 2018). Transstadial transmission is also essential for survival of *Rickettsia* spp. in ticks (Kelly and Mason, 1991).

In central Europe *I. ricinus* is the main vector and also reservoir of *R. helvetica* (Boretti et al., 2009; Sprong et al., 2009) and *R. monacensis* (Simser et al., 2002; Socolovschi et al., 2009b). Other *Rickettsia* spp. such *R. bellii*-like, *R. limoniae*-like (Tijsse-Klasen et al., 2011) and flea-borne/lice-borne *Rickettsia* (*R. typhi*, *R. prowazekii*) were also detected in *I. ricinus*, the latter probably as a result of cross-infection due to sharing rodents as reservoir hosts by ticks and fleas (Sprong et al., 2009). Recently, *Rickettsia felis* (causative agent of flea-borne spotted fever), strain “Danube” was isolated from a questing *I. ricinus* nymph in Slovakia (Danchenko et al., 2022).

*Anaplasma phagocytophilum* (order Rickettsiales, family Anaplasmataceae) is a Gram-negative obligate intracellular bacterium of medical and veterinary importance (Dumler et al., 2001). It invades white blood cells (predominantly neutrophils) and in humans, it causes human granulocytic anaplasmosis (HGA) (Blanco and Oteo, 2002), in ruminants it causes tick-borne fever (Woldehiwet, 1983). The main vectors are *I. ricinus* in Europe*, I. pacificus* and *I. scapularis* in Northern America, and *I. persculcatus* in Asia (Stuen et al., 2013). The reservoir hosts of the human pathogenic *A. phagocytophilum* strain in Northern America are mainly rodents. In Europe, the ecology of the bacterium is more complex and involves multiple strains associated with a great variety of vertebrate hosts, but the reservoir(s) of the human pathogenic strains have not been reliably defined (Stuen et al., 2013; Jahfari et al., 2014; Jaarsma et al., 2019). The infection in ticks is maintained through transstadial transmission, with nymphal and adult ticks infecting humans (Stuen et al., 2013).

*Neoehrlichia mikurensis* (family Anaplasmataceae) is an emerging tick-borne pathogenic bacterium occurring in Europe and Asia. In 1999, it was detected for the first time in *I. ricinus* from the Netherlands (Schouls et al., 1999) and was isolated in culture by Wass et al. (2019). The bacterium causes febrile disease mainly in immunocompromised human patients (Wennerås, 2015). The main vector of *N. mikurensis* in Europe is *I. ricinus* and rodents are suggested as its main reservoirs (Portillo et al., 2018). The bacterium is maintained in tick populations mainly transstadially, but transovarial transmission is suggested as well (Ondruš et al., 2020a).

Chlamydiae DNA was detected in *I. ricinus* ticks from Switzerland (Pilloux et al., 2015). Subsequently, a novel putative species, “*Candidatus* Rhabdochlamydia helvetica” was identified (Pillonel et al., 2019), however, it is still not clear if chlamydiae detected in ticks are pathogenic to mammals, tick endosymbionts, or tick parasites.

### 3.3 Apicomplexa

*Babesia* species (Piroplasmida, Babesiidae) are intraerythrocytic parasites that cause babesiosis in humans and animals (Yabsley and Shock, 2013). *Babesia* parasites undergo developmental changes in their vectors and vertebrate hosts: sexual phase (gamogony) in the midgut and asexual proliferation (sporogony) in salivary glands of ticks, and asexual reproduction (schizogony, merogony) in vertebrate erythrocytes (Jalovecka et al., 2018; Jalovecka et al., 2019). Transtadial transmission in *Babesia* is enabled by dormant sporoblasts present inside salivary gland cells. Transovarial transmission of *Babesia* in tick females occurs by the invasion of kinetes to the ovaries and is considered as a unique survival strategy in *Babesia* sensu stricto (Clade X, e.g. *B. divergens*, *B. venatorum, B. canis*) (Jalovecka et al., 2018; Jalovecka et al., 2019).

*I. ricinus* is the main vector of zoonotic *Babesia* spp. in Europe, namely *Babesia divergens*, *B. microti* and *B. venatorum* (Hildebrandt et al., 2021). Cattle and red deer are natural reservoirs of *B. divergens*, cervids (primarily roe deer) for *B. venatorum* and small rodents for *B. microti* (Yabsley and Shock, 2013). In addition to zoonotic species, *Babesia* sp. deer clade (*B.* cf. *odocoilei*) and *B. capreoli* causing asymptomatic infections in free ranging cervids have also been detected in *I. ricinus* (Azagi et al., 2021; Bajer and Dwużnik-Szarek, 2021) (see also **Supplementary Table 3**).

*Dermacentor reticulatus* is the main vector of *Babesia canis*, but the presence of this parasite was also detected in all active stages of questing *I. ricinus* (Liberska et al., 2021) suggesting transovarial and transstadial transmission of the parasite in *I. ricinus* populations. Another species associated with canines, particularly with the red fox, is *Babesia vulpes. Ixodes hexagonus* is probably its main vector, but the species was detected also in *I. ricinus* (Checa et al., 2018). However, the vector competence of *I. ricinus* for *B. canis* and *B. vulpes* has not been confirmed and needs further investigations (Bajer and Dwużnik-Szarek, 2021).

In spite of extensive information on their epidemiological importance, knowledge of molecular interactions of piroplasmids with their tick vectors, particularly *I. ricinus*, is limited (Antunes et al., 2017; Jalovecka et al., 2019). A few tick molecules potentially involved in piroplasmid acquisition, propagation and transmission by ticks through their saliva have been determined mainly in studies on *Rhipicephalus microplus* and *Haemaphysalis longicornis* (Antunes et al., 2017). They include molecules enabling the parasites to penetrate the tick midgut perithrophic membrane and invade epithelial cells (e.g. TROSPA), molecules involved in regulating *Babesia* infection (defensins and antimicrobial peptides – longicin, microplusin, longipain, LRR-domain and Kunitz-type protease inhibitors, Bm86, subolesin), invasion of other tick organs such as ovaries (calreticulin, glutamine synthetase, Kunitz-type serine protease inhibitors, vitellogenin receptor) and salivary glands (calreticulin, TROSPA).

*Theileria* spp. (Piroplasmida, Theileriidae) infect lymphocytes, erythrocytes and other cells of the internal organs of a variety of vertebrate hosts. In contrast to *Babesia*, they are characterized by schizogony in nucleated blood cells (monocytes, lymphocytes) and subsequent invasion of erythrocytes. In Europe, asymptomatic infections caused by *Theileria* spp. are known in free-living cervids and caprines, but none of these species has been found to cause zoonotic disease (Yabsley and Shock, 2013). Occurrence of *Theileria* spp. phylogenetically related with *T. capreoli* and *Theileria* sp. OT3 have been reported from *I. ricinus* feeding on infected cervids (Hamšíková et al., 2016a; Fernández et al., 2022), but *I. ricinus* is probably not their vector.

*Hepatozoon* spp. (Eucoccidiorida, Hepatozoidae) are intraerythrocytic parasites of different vertebrates. *Hepatozoon canis* DNA has been sporadically detected in questing *I. ricinus* (Hamšíková et al., 2016b) and in *I. ricinus* removed from dogs (Ciuca et al., 2021). However, the vector role of this tick species for *H. canis* has not been confirmed.

*Toxoplasma gondii* (Eucoccidiorida, Sarcocystidae) is an obligate intracellular parasite that causes toxoplasmosis in humans and animals and is capable of infecting all species of warm-blooded animals. Only Felidae are known as definitive hosts of the parasite and produce oocysts in their feces which develop into infectious sporozoites. Oral infection through contaminated food or water is considered the main route of infection. Congenital transmission is also possible. However, due to the wide range of infected hosts, other paths of infections have been suggested, including transmission through blood-feeding arthropods. By examining questing and host feeding *I. ricinus* ticks in Poland, over 10% of questing ticks were found infected suggesting that ticks may be involved in the spread of toxoplasmosis (Adamska and Skotarczak, 2017).

### 3.4 Kinetoplastea

*Trypanosoma* species (Trypanosomatida, Trypanosomatidae) is a group of parasitic flagellate protozoa transmitted by hematophagous insects (dipterans, heteropterans, fleas) causing serious diseases in humans and animals. Presence of trypanosomes has previously been detected also in different species of questing ticks, including *I. ricinus* (Rehacek et al., 1974). Recently, *Trypanosoma* sp. Bratislava1, was isolated from questing *I. ricinus* adults collected in Slovakia in tick cell culture and partially characterised (Luu et al., 2020). This may be a new species related to species detected in ticks in South America and Asia, and to *Trypanosoma caninum* isolated from dogs in Brazil. However, information about reservoirs, routes of transmission and pathogenicity of the new species are missing.

### 3.5 Microsporidia

Microsporidia is a large group of obligate, intracellular spore forming eukaryotic parasites. Seventeen species have been associated with human diseases (Han and Weiss, 2017). However, ticks are probably not vectors of zoonotic microsporidia (Trzebny et al., 2022), but they can occasionally be found infected with these parasites. In general, knowledge on microsporidial infections in ticks is limited and only three species have been detected in *I. ricinus* by microscopic methods: *Nosema slovaca* and *Unikaryon (Nosema) ixodis* in former Czechoslovakia by Weiser and Rehácek (1975) and Weiser et al. (1999) and a *Nosema*-like species in Moldova by Tokarev Iu and Movilé (2004). Recently, metabarcoding revealed low prevalence of microsporidian parasites in questing *I. ricinus* and in those collected from dogs in Poland. *Encephalitozoon intestinalis*, a potentially zoonotic species was detected in ticks that fed on dogs and a potentially new *Endoreticulatus* species in questing ticks (Trzebny et al., 2022). *Nosema slovaca* was also detected and isolated from *D. reticulatus* collected in Hungary. Laboratory infection of partially fed *D. reticulatus* females by the *Nosema* isolate caused an acute infection and death of the ticks. Thus, microsporidia could be ranked among potential biological control agents of ixodid ticks (Řeháček et al., 1996).

### 3.6 Fungi

In contrast to insects (Chakraborty et al., 2020; Višňovská et al., 2020; Kim et al., 2021a; Guo et al., 2022), the research on associations between fungi and ticks is limited. The majority of the available studies are focused on entomopathogenic fungi, mainly of the *Beauveria* and *Metarhizium* genera, and their prospective use in the biological control of ticks (Kalsbeek et al., 1995; Mitina et al., 2011; Munteanu et al., 2014; Wassermann et al., 2016). Entomopathogenic fungi invade ticks in the soil and leaf litter where development of ticks takes place. *Beauveria* and *Metarhizium* species were identified, for example, in adult *I. ricinus* and *D. reticulatus* from Poland and majority of the fungal isolates proved to be effective against both tick species (Szczepańska et al., 2020). Other fungi that could potentially be considered as biological control agents are contaminants found in laboratory tick colonies. Three different taxa have been isolated recently from *I. ricinus* colonies, *Penicillium steckii*, *Aspergillus parasiticus* and *Scopulariopsis brevicaulis* (Bonnet et al., 2021) (**Supplementary Table 3**).

### 3.7 Nematodes

*I. ricinus* ticks in nature were found parasitized by larvae of species belonging to the family Mermithidae (Lipa et al., 1997). *I. ricinus* is the intermediate host and vector of *Cercopithifilaria rugosicauda* (Spirurida, Onchocercidae), a subcutaneous filarial parasite of the European roe deer (Winkhardt, 1980). Nematodes from families Steinernematidae and Heterorhabditidae, are acaropahtogens, and are mainly of interest as tick biocontrol agents (Samish and Glazer, 2001; Hartelt et al., 2008) (see also **Supplementary Table 3**).

## 4 Microbiome in tick organs

The localization of symbionts in certain organs seems to be associated with two factors – nutritional needs and reproduction. Endosymbionts colonize specific tissues most likely due to nutrient availability and to ensure its survival and transmission. As mentioned earlier, localization of endosymbionts in tick Malpighian tubules is most likely tied to nutrient availability. Ticks’ metabolic waste concentrated in Malpighian tubules can be recycled by its endosymbiotic bacteria that produce necessary nutrients for the ticks, such as B vitamins (Reinhardt et al., 1972; Klyachko et al., 2007; Azagi et al., 2017; Zhang et al., 2022). The majority of endosymbionts can be found in ovarian tissues, including *Francisella*-LE, *Coxiella*-LE, *Rickettsia* endosymbionts, or *Cand.* Midichloria mitochondrii (Liu et al., 2016; Buysse et al., 2019; Olivieri et al., 2019). *Coxiella*-LE and *Francisella*-LE are capable of producing vitamin B2, B6 and B9. Increased requirements for micronutrients and macronutrient after female feeding, with subsequent oogenesis, may demand immediate and locally available source. Ovaries, and subsequently oocytes, represent a means for reproduction and transmission of the endosymbionts. Their prevalence also rapidly increases following blood-feeding (engorgement), and peaks during oogenesis due to the influx of nutrients (Sassera et al., 2008; Zhang et al., 2022). This increase in the proliferation of symbionts in fed females can supply the necessary nutrients and energy for oogenesis (Zhang et al., 2022). The microbiome of salivary glands (SG) is mostly associated with the presence of tick-borne pathogens, such as *B. burgdorferi* s.l., *Rickettsia* spp., or *A. phagocytophylum*. SG along with tick saliva enhance transmission of tick-borne pathogens to the vertebrate host during the tick feeding period (Liu et al., 2011; Lejal et al., 2019; Helble et al., 2021). Since most tick endosymbionts are closely related to tick-borne pathogens, their occurrence in SG can indicate their possible pathogenicity for a vertebrate host. However, only very scarce evidence on the transmission of such bacteria, e.g. *Cand.* M. mitochondrii from ticks SG to the vertebrate hosts blood (Cafiso et al., 2019; Sgroi, 2022) or presence of antibodies in human sera samples after tick engorgement (Mariconti, 2012) has been found so far. *Rickettsia* endosymbionts, *Coxiella*-LE or *Francisella*-LE are commonly found in SG. They are producents of B vitamins an possibly play a nutritional role in ticks’ diet. Vitamin B2 can be synthetized only by plants or microorganisms and eukyrotes cannot synthetise vitamin B6 at all. Thus, for ticks the only source of essential micronutrients are the bacteria which provide the host with the ability to metabolize macronutrients and produce energy (ATP). Moreover, vitamin B6 is also important for the metabolism of amino acids, neurotransmitters and nucleic acids.

### 4.1 Digestive system

For blood-feeding arthropods, blood is fundamental source of nutrients and is crucial for development – oogenesis and moulting (Brunner et al., 2011; Valzania et al., 2019). Arthropods with a diet primarily consisting of blood commonly possess a microbiota in their alimentary tract that influences their vector competence for pathogens (Dong et al., 2009; Cirimotich et al., 2011). Introduction of blood into the digestive system commonly leads to the multiplication of microbial community in the midgut of hematophagous arthropods, such as mosquitoes (Oliveira et al., 2011b). Ixodid ticks increase in size 100 to 1000 times during feeding on a vertebrate host due to the immense influx of blood which represents a nutritionally rich substrate and is possibly available also for microbiota present in the tick midgut. In contrast to other hematophagous arthropods whose digestion of blood is an extracellular process, ticks digest blood intracellularly in the functional midgut cells that are able to perform endocytosis (see above). It was also found that, in contrast to other blood-feeding arthropods, blood intake by ixodid ticks is not associated with growth of microbiome in the midgut (Ross et al., 2018; Guizzo et al., 2020). In fact, diversity and quantity of midgut bacteria of *I. ricinus* decrease after blood meal. There are various hypotheses explaining this phenomenon. According to the first hypothesis, hemoglobin fragments and complement system from host blood may have negative impact on midgut bacteria and are responsible for their decline (Fogaça et al., 1999; Nakajima et al., 2003; Sforça et al., 2005). Another hypothesis says that the tick digestive cells reduce midgut microbiota. During blood digestion, midgut bacteria might be together with blood elements endocytosed and digested by tick digestive cells (Lara et al., 2005). According to the third hypothesis, tick immunity affects the population of bacteria in the tick midgut by antimicrobial peptides and reactive oxygen species (ROS). *I. ricinus* midgut transcriptome shows that genes associated with immunity, such as lysozyme and defensin, were considerably upregulated during tick feeding (Isogai et al., 2009; Perner et al., 2016).

*I. ricinus* midgut was found to harbor a fluctuating bacterial population whose compositions differs in individual ticks, depending on the environment and/or infection with pathogens. Detection of bacterial taxa, such as *Neisseria, Prevotella* and *Staphylococcus* in midgut of *I. ricinus* and other tick species indicates that the host skin is the potential source of these bacteria. But most of bacteria identified in the midgut originated from the environment. Wild ticks have higher microbiome diversity compared to ticks raised in laboratory conditions (Dimitriu et al., 2019; Guizzo et al., 2020; Kumar et al., 2022b). Other commonly found bacteria in tick midgut are tick-borne pathogens, such as *Borrelia* spp. and *Rickettsia* spp. *Borrelia* spp. enter the tick mostly during feeding on an infected host, through blood, in early life stages and persist in the midgut throughout the rest of tick’s development, although *B. miyamotoi* can be transmitted also transovarially. Thanks to evolution of unique metabolic strategies, *B. burgdorferi* s. l. can persist in thiamine-limited environment of the tick midgut. Moreover, *B. burgdorferi* s. l. can outcompete other bacteria for limited nutrients and dominate the tick midgut microbiome (Benach et al., 1987; Ross et al., 2018). As no common bacterial genera of tick midgut were found amongst individual ticks, it is possible to conclude that tick midgut does not harbor a stable, core microbiome (Guizzo et al., 2020). However, studies on the microbiome of *I. ricinus* found large bacterial diversities, an overall unstable microbial composition, and an extremely low bacterial load in their midgut (Aivelo et al., 2019). The observations made by Guizzo et al. (2020) that *I. ricinus* midgut microbiome has a relatively high diversity but is low in abundance, except for tick-borne pathogens (genus *Borrelia*) and endosymbionts (*Spiroplasma* or *Rickettsia*) are in line with findings of Lejal et al. (2019). Furthermore, it has been shown that the bacterial composition in the *I. ricinus* is dynamic from temporal as well as geographical point of view. It is influenced also by life stage associated with feeding on different hosts as well as different environment. The majority of these environmentally gained bacteria are relatively quickly eliminated and are unable to colonize the gut (Lejal et al., 2019; Krawczyk et al., 2022).

### 4.2 Ovaries

Contrasting the midgut, tick ovaries harbor a stable microbial community, mostly of low diversity. Ovaries of *I. ricinus* are dominantly inhabited by *Cand.* Midichloria spp. endosymbionts (Sassera et al., 2008; Olivieri et al., 2019; Guizzo et al., 2020). Other dominant ovarian endosymbionts belong to the genus *Coxiella*. For example, *Coxiella* spp. endosymbionts in the ovaries of *Rhipicephalus microplus* were found to represent over 98% of the microbiome population (Andreotti et al., 2011). *Rickettsia* spp., *Francisella* spp. and *Wolbachia* spp. are also common in the ovaries of *Ixodes* spp., *Rhipicephalus* spp., *Haemaphysalis* spp. and *Hyalomma* spp. (Noda et al., 1997; Kurtti et al., 2015; Azagi et al., 2017).

### 4.3 Salivary glands

Salivary glands and saliva play an important role in transmission of most tick-borne pathogens to the vertebrate host. It is widely accepted that pathogens, such as *B. burgdorferi* s. l., mainly reproduce in tick gut and only migrate to SG at the beginning of the blood meal (des Vignes et al., 2001; Sertour et al., 2018) (see also in Section 3 on pathogens). However, Sertour et al. (2018) detected *B. burgdorferi* s. l. in SG of *I. ricinus* also prior to blood meal. Persistence of these bacteria in the SG of unfed ticks might suggest that this organ could serve as their potential reservoir (Lejal et al., 2019). In addition to *B. burgdorferi* s. l., *Rickettsia* spp., *Anaplasma* spp. and *Neoehrlichia mikurensis* were also detected in SG of *I*. *ricinus* (Lejal et al., 2019; Ondruš et al., 2020b). For comparison, the endosymbiont *R. buchneri* was found to colonize SG of *I. scapularis* (Al-Khafaji et al., 2020) and a *Coxiella*-like endosymbiont was observed in the granular acini of SG of *Amblyomma americanum* (Klyachko et al., 2007).

Di Venere et al. (2015) focused on proteomic analysis in ovaries and SG of *I. ricinus* and its endosymbiont *Cand*. Midichloria mitochondrii and created proteomic profiles of SG and ovaries. In similar manner, Cotté et al. (2014) investigated expression of proteins in *I. ricinus* SG in the presence of *B. burgdorferi* s. l. Presence of 12 of 120 identified proteins was modulated by *B. burgdorferi* s. l., most of which were upregulated and are involved in protein synthesis and cell defence. Liu et al. (2011) studied P11 protein secreted from *I. ricinus* SG which is important for migration of *A. phagocytophilum* from gut to SG.

Kim et al. (2021a) discovered that nymphs of *I. scapularis* infected with *B. burgdorferi* s. l. secreted significantly more saliva proteins while suppressing antimicrobial peptides up to 85-fold compared to uninfected ticks. *Borrelia burgdorferi* s. l. regulated protein composition in tick saliva to retain its survival at the tick feeding site. Saliva proteins that were upregulated in infected ticks most likely play a significant role in transmission and survival of the spirochaetes. An example of upregulated proteins is thioredoxin that neutralizes oxygen peroxide, which is highly toxic for *B. burgdorferi*, or pyruvate kinase that is required for pyruvate production, which protects the spirochaetes from effects of oxygen peroxide. On the contrary, saliva proteins that were suppressed in *B. burgdorferi*-infected ticks, e.g., copper/zinc superoxide dismutase that leads to production of oxygen peroxide, are probably harmful or hinder transmission of *B. burgdorferi* (Kim et al., 2021b).

*Anaplasma phagocytophilum* infecting SG of *I. scapularis* facilitates the infections by inhibiting the intrinsic apoptosis pathway. Tick cells are able to limit infections with the extrinsic apoptosis pathway while increasing feeding and survival (Ayllón et al., 2015; Villar et al., 2015; de la Fuente et al., 2016; Cabezas-Cruz et al., 2017).

## 5 Tick-microbiome relationships

### 5.1 Interactions of bacteria within the tick microbiome

Compared to other groups of arthropods, ticks host and transmit a greater diversity of pathogens. Majority of tick-borne pathogens are generally considered separately from the rest of the tick microbiome. Nonetheless, as they are able to reproduce within ticks, survive tick moulting and some can be transmitted transovarially, they can be considered members of the tick microbiome (Pollet et al., 2020).

For example, association between *Rickettsia* spp. and *Cand.* Midichloria mitochondrii was observed in *I. ricinus*. *Cand.* Midichloria mitochondrii abundance was significantly higher in *Rickettsia*-infected ticks suggesting that *Cand.* Midichloria facilitates colonisation of *I. ricinus* by *Rickettsia* spp. (Lejal et al., 2021). Similar interaction was observed in the tick *Amblyomma maculatum*, where in both unfed and fed females the quantity of *Cand.* Midichloria mitochondrii was notably higher in organs (ovaries, midgut and SG) of ticks infected with *Rickettsia parkeri* than in *R. parkeri*-free ticks. The same study also showed that in midgut of the females infected with *R. parkeri* reduced levels of *Francisella*-like endosymbionts were detected (Budachetri et al., 2018).

There are contradictory reports regarding the impact of *Borrelia* spp. on the tick microbial community. In earlier reports, *I. scapularis* females infected with rickettsial endosymbionts had notably smaller rates of *B. burgdorferi* s. l. infections in comparison to males that were lacking this symbiont (Steiner et al., 2008). *Ixodes pacificus* nymphs infected with *B. burgdorferi* s. l. had lower microbiome diversity than uninfected ticks, indicating that pathogen infection might be associated with microbiome diversity (Kwan et al., 2017). In contrast, no substantial differences in microbiome diversity of *B. burgdorferi* s. l. infected and uninfected *I*. *pacificus* ticks were observed by (Swei and Kwan, 2017). This observation was confirmed by Brinkerhoff et al. (2020) and Chauhan et al. (2019), who analyzed *I. scapularis* ticks infected with *B. burgdorferi* s. l. and did not find any association between diversity of tick microbiome and infections with *B. burgdorferi*. Though, these studies showed a connection between presence of certain bacterial taxa and their quantity, and *B*. *burgdorferi* (Chauhan et al., 2019; Brinkerhoff et al., 2020). Ticks infected with *B*. *burgdorferi* had higher amount of *Cutibacterium* spp. and lower number of species belonging to Beijerinckiaceae and Diplorickettsiaceae families, and bacteria of genus *Rickettsia* in their microbiome (Chauhan et al., 2019). It was also demonstrated that presence of bacterial genera such as *Tepidomonas, Francisella* and *Fibriimonas* were associated with the presence of *B. burgdorferi* s. l. (Brinkerhoff et al., 2020).

In *I. ricinus*, presence of human pathogenic genospecies of *B. burgdorferi* s. l. was found to shift the abundances of *Cand.* Midichloria, *Rickettsia*, *Pseudomonas*, *Staphylococcus*, and *Cand*. Neoehrlichia in the tick microbiome. The geographic location was less important in the tick microbiome composition but shifted the abundances of *Pseudomonas* and *Wolbachia* (Hoffmann et al., 2021). Another study has proved that tick larval dysbiosis after surface sterilization of eggs did not affect vector competence of *I. ricinus* for *B. afzelii* in the laboratory and the effect of egg surface sterilization on the tick bacterial microbiome disappeared in the moulted nymphs. However, the bacterial microbiome of *I. ricinus* nymphs that fed as larvae on *Borrelia*-infected mice was less abundant but more diverse than in nymphs fed on uninfected animals showing that infections in the vertebrate hosts can alter the microbiome of arthropod vectors (Hamilton et al., 2021).

### 5.2 Microbiota and tick behavior

Thanks to coevolution, ticks and tick-borne pathogens have created an intimate relationship. Physiological changes in pathogen-infected ticks modify *Ixodes* spp. behaviour in different ways (Benelli, 2020): changing mobility and questing activity (Alekseev et al., 2000; Faulde and Robbins, 2008; Kagemann and Clay, 2013), acquisition of bloodmeal (Dai et al., 2010; Narasimhan et al., 2014), modification of saliva production (Pham et al., 2021), or protection against unfavourable environmental conditions (Lefcort and Durden, 1996; Oliver et al., 2010; Ferrari and Vavre, 2011; Romashchenko et al., 2012).

### 5.3 Microbiota and tick´s vitamin supplementation

**Figure 2:**
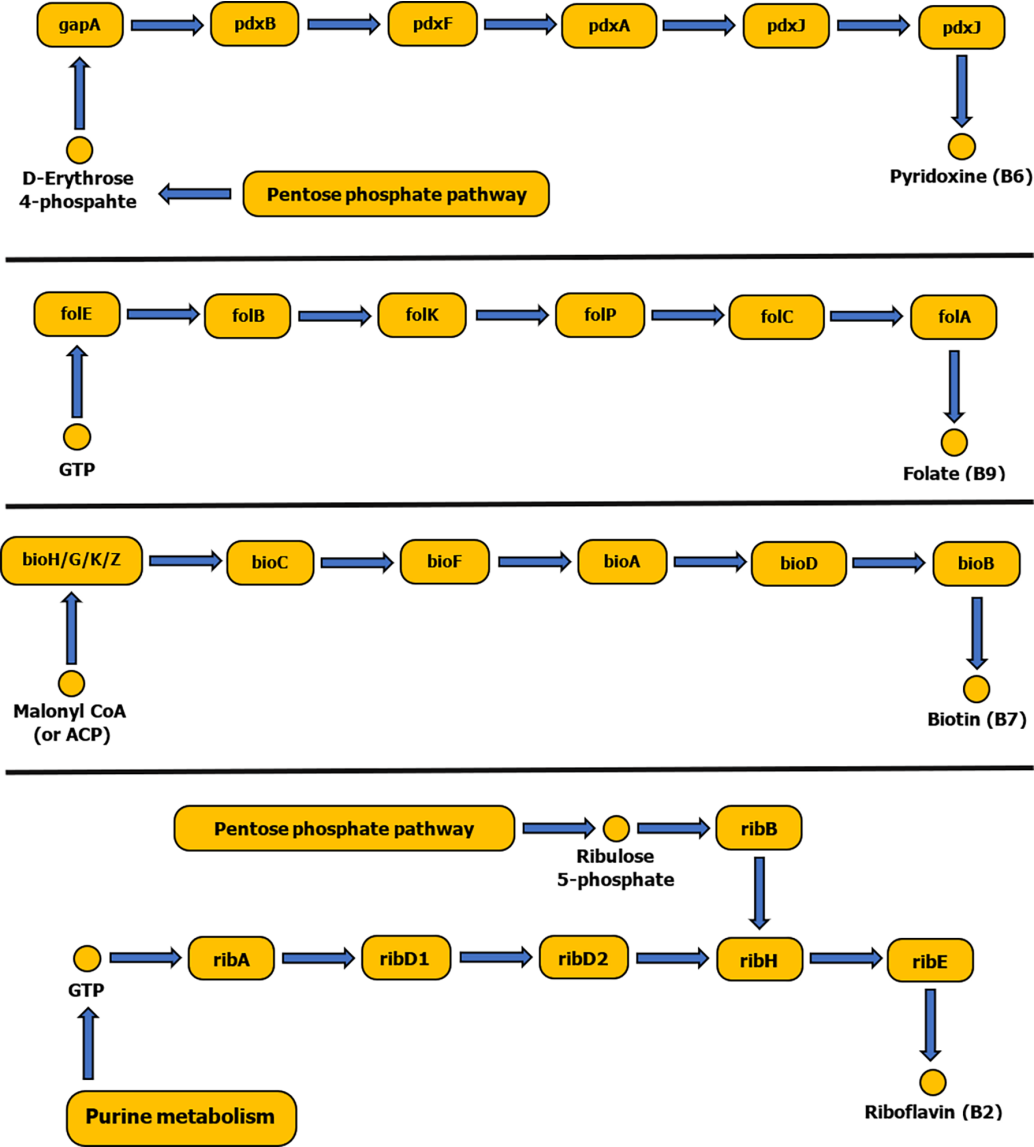
Schematic diagram showing biosynthetic pathways of vitamins B6, B9, B7 and B2. Pathways were constructed based on the KEGG reference database and pathways diagrams available in RAST SEED viewer.

The tick symbiotic microbiome consists of relatively diverse species that form a comprehensive symbiotic system offering a nutritional adaptation for a constrained tick diet. Vertebrate blood is their exclusive source of food regardless of its nutritious imbalance. The tick genome includes genes related to heme and hemoglobin digestions, iron metabolism, osmotic homeostasis and vitamin shortage, but lacks genes for biosynthesis of essential vitamins (Jia et al., 2020; Buysse et al., 2021). The tick microbiome is dominated by intracellular bacterial symbionts that complement this vitamin deficiency (**Figure 2**). They possess genes for biosynthesis of a fundamental set of B vitamins: B7 (biotin), B2 (riboflavin) and B9 (folate) (Buysse et al., 2021).

**Figure 3:**
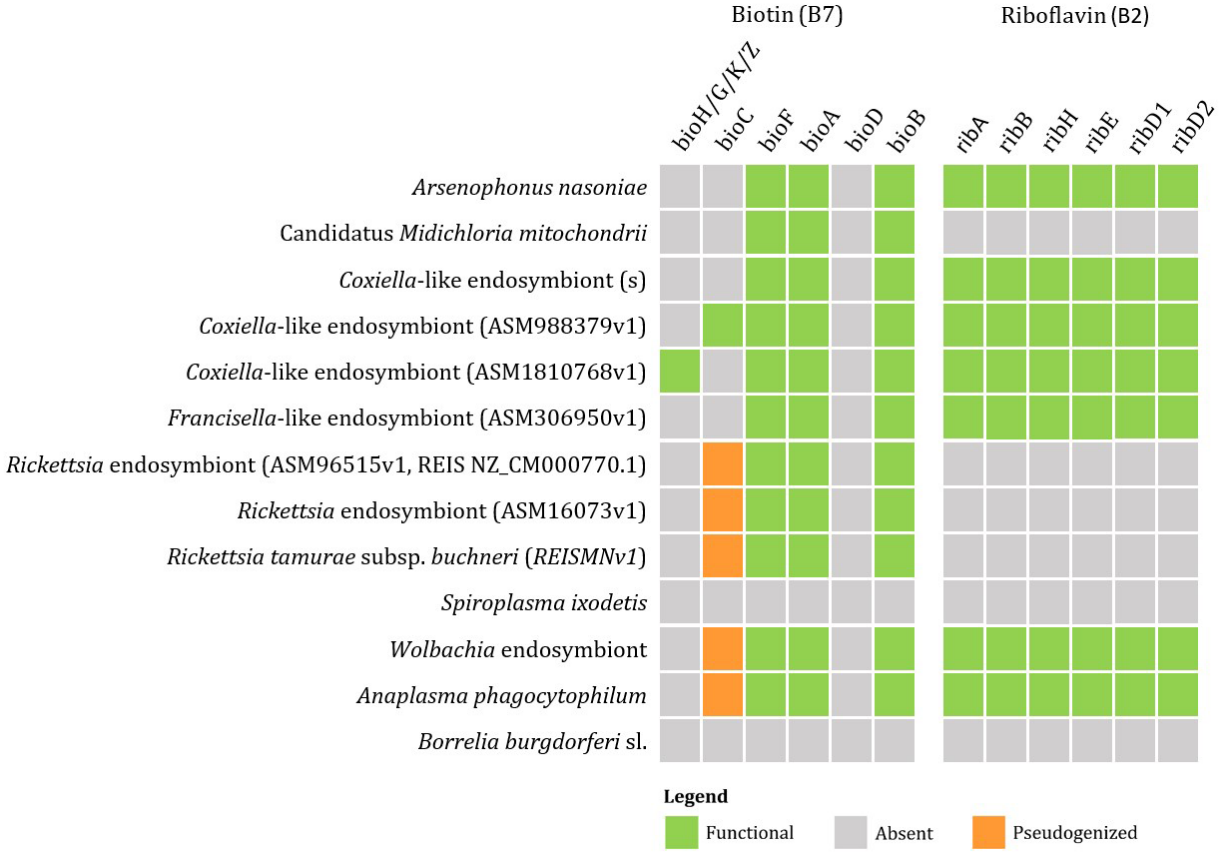
Schematic overview of endosymbiotic bacteria and pathogens transmitted by ticks with focus on gene clusters involved in vitamin B production (B7, B2). Color code: green – detected genes, grey – absent genes, orange – pseudogenized genes.

**Figure 4:**
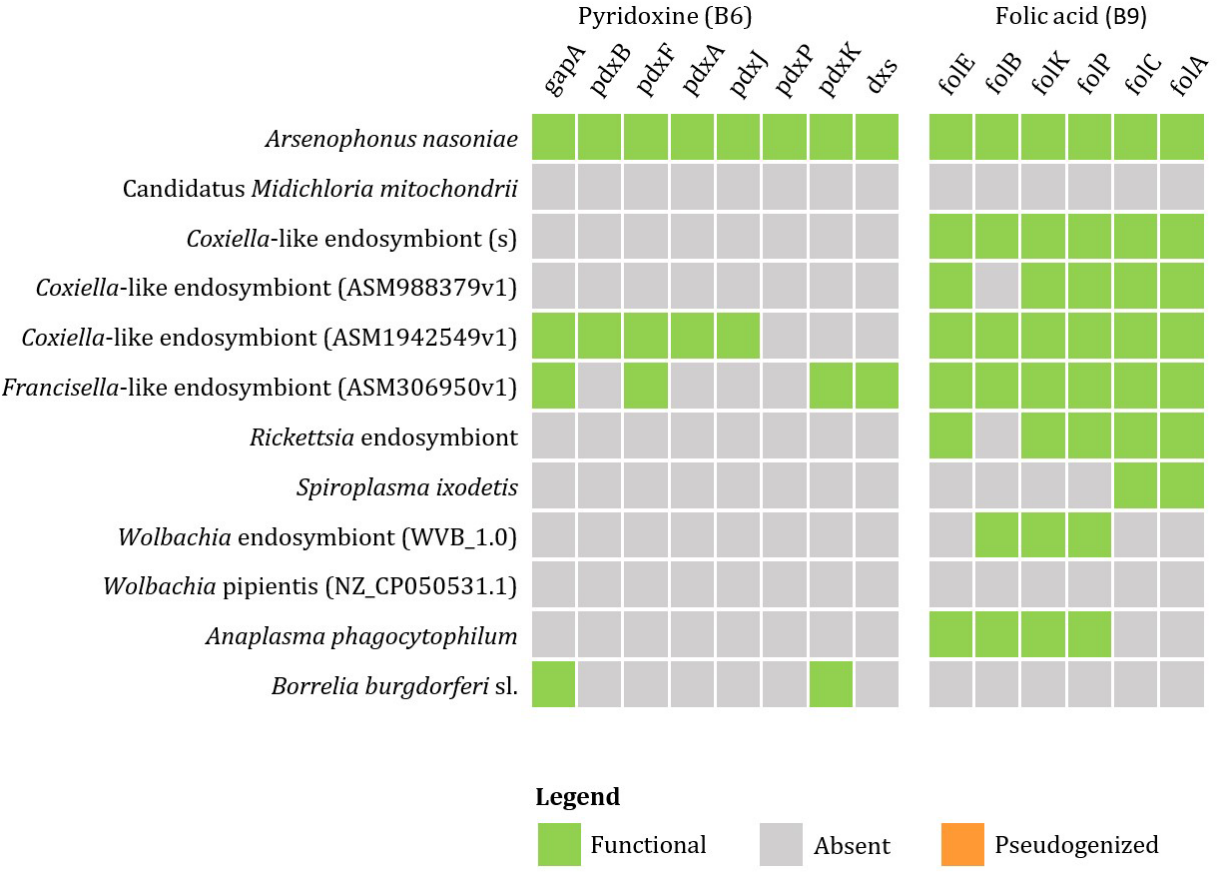
Schematic overview of endosymbiotic bacteria and pathogens transmitted by ticks with focus on gene clusters involved in vitamin B production (B6, B9). Color code: green – detected genes, grey – absent genes, orange – pseudogenized genes).

Currently available genomic data of the representatives of tick endosymbionts and selected pathogens from GenBank show that tick-associated endosymbionts of the genus *Rickettsia* were found to be mainly producers of vitamin B9 (folate) with exception of *R. buchneri*, which also possesses genes for biosynthesis of vitamin B7 (biotin). *Coxiella*-like endosymbionts, *Francisella*-like endosymbionts and *A. nasoniae* were found to be the most beneficial bacteria in terms of vitamin B production. Both, *Coxiella*-like and *Francisella*-like endosymbionts possess complete metabolic pathways for biosynthesis of biotin, folate and vitamin B2 (riboflavin), while *A. nasoniae* is able to produce vitamin B6 (pyridoxine) (**Figures 3**, **4**).

**Figure 5:**
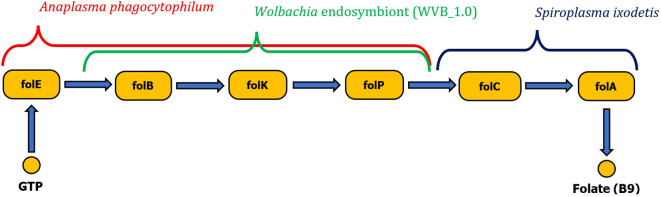
Schematic visualization of complete folate biosynthesis pathway formed by two gene clusters originating from either *Wolbachia* endosymbiont or *Anaplasma phagocytophilum* pathogen complementary with the *Spiroplasma ixodetis* gene cluster (*folC*, *folA*).

*Wolbachia* sp. and *A. phagocytophilum* were found to possess only the first half of the folate biosynthetic pathway (folB to folP). However, the complexity of the metabolic pathway for folate biosynthesis can be provided by coinfection with *S. ixodetis*, which possesses the second half of the folate biosynthesis pathway (enzymes folC and DHFR). *Anaplasma phagocytophilum* also possesses complete pathways for biosynthesis of biotin and riboflavin (**Figure 5**). No genes for production of vitamin B have been identified in *B. burgdorferi* s. l. associated with *I. ricinus*.

### 5.4 Microbiota and energetic metabolism

Whole-genome analysis of *Cand.* Midichloria mitochondrii confirmed the presence of cbb_3_ cytochrome oxidase. This cytochrome oxidase belongs to the C-family of the HCO (hem-copper oxidase) superfamily and has been described in proteobacteria (Myllykallio and Liebl, 2000). Compared to A- and B-family HCOs, the cbb_3_ has a higher affinity for O_2_ reduction, but is less efficient (Sassera et al., 2011). Oogenesis in ticks has high oxygen requirements (Aboul-Nasr and Bassal, 1972) that result in oxygen concentrations that are suboptimal for mitochondrion ATP synthesis. The presence of cbb_3_ oxidase, which is expressed only under these microanaerobic conditions, might enable *Cand.* Midichloria mitochondrii to synthesize ATP. As *Cand.* Midichloria mitochondrii is localized in ovaries, there is a possibility, that it may serve as a supplementary ATP source for host cells during oogenesis (Sassera et al., 2011). Regarding the possible impact of *Cand. Midichloria* mitochondrii on eggs development or supplementation of the needs of the eggs during their development has not been investigated so far.

*In silico* analysis of tick *Coxiella-*LE revealed the presence of a gene for deoxyhypusine synthase (DHPS). DHPS is required for the activation of eukaryotic initiation factor 5A (elF5A) with hypusine (Wolff et al., 1995). Hypusinated elF5A (elF5A^H^) affects mitochondrial respiration by promoting the efficient expression of a subset of mitochondrial proteins involved in the TCA cycle and oxidative phosphorylation (Puleston et al., 2019; Liang et al., 2021). Regulation of host mitochondria by *Coxiella*-LE is already known by mitochondrially-targeted effector proteins (Fielden et al., 2021) and the production of DHPS may serve as another tool for *Coxiella* spp. to modulate mitochondria function.

### 5.5 Microbiota and tick´s response to stress

In *I. ricinus* ticks infected with *B. burgdorferi* s. l. reduced mobility and higher tendency to stay immobile, rather than to move towards more humid environment was observed, which is more advantageous for keeping tick water balance (Herrmann and Gern, 2012). Infected ticks have increased survival under challenging thermohydrometric conditions. The pathogen is likely to change metabolism or physiology of tick organs that are involved in water regulation (Herrmann and Gern, 2010). It has also been observed that *B. burgdorferi* s. l.-infected ticks have higher fat reserves (i.e. higher energy reserves) (Herrmann et al., 2013). The spirochetes probably possess the ability to change their gene expression according to temperature and also might be able to influence tick gene expression depending on thermal conditions (Ojaimi et al., 2003). Compared to uninfected *I. ricinus* ticks that commonly increase their movement during desiccating conditions, infected ticks tend to slow down their metabolism and keep questing longer. This gives the pathogen more time and thus more opportunities to find and infect a vertebrate host (Perret et al., 2003).

*Anaplasma phagocytophilum* infection impacts tick questing, feeding, survival and can manipulate expression of tick genes (Cabezas-Cruz et al., 2016; Cabezas-Cruz et al., 2017). In contrast to *B. burgdorferi* s. l., *A*. *phagocytophilum* infection was found to increase tick questing speed during challenging thermohydrometric conditions. In *A. phagocytophilum-*infected *I. scapularis* ticks, the pathogen induces synthesis of heat shock protein hsp20 and hsp70. These proteins are involved in response to heat stress, thus decreasing chance of desiccation and increasing tick survival rates (Busby et al., 2012). Furthermore, hsp20 with immunogenic properties is similar to myosin-like proteins that have been shown to accumulate after infection. Increase of two myosin subunits has been associated also with infection of *R. microplus* with *Babesia* and so seems to be connected with successful pathogen transmission (Kim et al., 2016).

*Anaplasma phagocytophilum* infecting *I. scapularis* activates expression of the *iafgp* gene that is coding the antifreeze glycoprotein IAFGP (*Ixodes scapularis* AntiFreeze Glycoprotein). This protein serves as biofilm formation inhibitor. IAFGP binds to bacterial peptidoglycan, altering permeability and ability to form biofilm. By upregulating *iafgp* and thus inhibiting biofilm development and altering gut microbiome, *A*. *phagocytophilum* is able more effectively colonise the tick (Abraham et al., 2017). Another function of IAFGP is cold protection. *Ixodes scapularis* infected with *A. phagocytophilum*, which induces IAFGP production, have improved cold tolerance and thus increased survival in a cold environment (Neelakanta et al., 2010).

Glycine-rich proteins (GRP) are found in many organisms and their function varies; they are involved in processes from structural to cellular level. In ticks, GRP in saliva possess a fundamental role in formation of tick cement which is also suggested to protect the tick from the host-associated immune factors. Still, there is little evidence that they are involved also in host defense and stress response. Overexpression of GRP can provide the organism with tolerance to stress including abiotic (cold, heat, draught) or biotic factors (microbial homeostasis, hematophagy). Stress stimuli, including oxidative stress, or injury cause significant upregulation of GRPs, however, mechanism of stress mediation is yet unknown (Bullard et al., 2019). Higher expression of GRP can improve the resistance of organism and can even enhance its survival. GRPs can affect the microbial population. Within the SG microbial community of *A. americanum* involvement of GRP AamerSigP-41539 was established as one of the microbial homeostasis maintenance factors. Some of GRPs, such as Attacins, are immune proteins with antimicrobial activity, but they might switch their role in case of different type of stress conditions, or may serve other functions during molting, fasting or overwintering (Bullard et al., 2019). In *Glossina morsitans*, Attacins play a role in *Trypanosoma resistance*, and can maintain homeostasis in infected individuals (Wang et al., 2008). Also, in the study of Flores-Ramirez et al. (2019) three glycine-rich proteins have been identified as a response to infection of *D. reticulatus* ticks that was experimentally infected with *Rickettsia slovaca*. Except of glycine-rich proteins, also glycine-proline rich proteins have been detected in ticks as a response to infection. They are associated with ticks’ attachment and feeding on the host, especially they are involved in host´s immune system evasion (Leal et al., 2018). Although the above mentioned proteins are involved in stress responses of other ticks or arthropods than *I. ricinus*, there is a probability that future studies will reveal at least some of them also in *I. ricinus*.

### 5.6 Microbiota and the tick immune system

The immune system of ticks consists of cellular and humoral responses (Sonenshine and Hynes, 2008). Hemocytes, the tick blood cells, are able to recognize, attack and phagocytose microorganisms entering the tick´s body cavity. After proliferation and differentiation, they represent an effective immune system of the tick that can deal with microbiota reaching the hemocoel (Kuhn, 1996). Humoral defence in ticks is secured by a variety of antimicrobial compounds, such as defensins, ixodidin or microplusin (Fogaça et al., 2006; Hajdušek et al., 2013). Phagocytosis of invading microbes by tick hemocytes is most likely facilitated by a complement-like system composed of various lectins, thioesters, protease inhibitors, convertases or iron-binding proteins. Hemocyte-expressed thioester-containing proteins IrTEPs (*I. ricinus* thioester-containing proteins) were found to mediate phagocytosis of the Gram-negative bacterium *Chryseobacterium indologenes* and to lesser extent phagocytosis of yeast *Candida albicans* (Urbanová et al., 2015).

The protease inhibitor of the α2-macroglobulin family from *I. ricinus* (IrAm) and *I. ricinus* factor C2/factor B (IrC2/Bf) presence can be found in ticks hemolymph. Inactivation of IrAM significantly reduces the capability of hemocytes to phagocytise *C. indologenes*, however *B. burgorferi* was not affected. The specificity of IrAM may be linked to the metalloprotease of *C. indologenes* (Buresova et al., 2009). In contrast, the presence of *Borrelia* sp. and *C. albicans* significantly upregulated the expression if IrC2/Bf. Silencing of IrC2/Bf inhibited phagocytosis of *Borrelia* and *C. albicans* by ticks’ hemocytes. IrC2/Bf most likely functions as a convertase in the complement inactivation pathway leading to the elimination of *Borrelia* and yeast infection (Urbanová et al., 2018). Since iron is an indispensable element for most organisms, iron management is an important component of innate immunity. Hemocyte-produced ferritins of *Haemaphysalis longicornis* (HlFERs) take part in cellular immune response, most likely through their function of iron-sequestration. Infection of ticks by *E. coli* resulted in stimulation of HlFERs expression in hemocytes and silencing of HlFERs resulted in a significantly lower survival rate of infected ticks (Galay et al., 2016). Other important immune proteins are fibrinogen related proteins, Ixoderins. Some Ixoderins serve as opsonin’s in the tick hemolymph aiding phagocytosis. Ixoderin A (ixo-A) was found to be expressed in hemocytes and is most likely responsible for the hemagglutination activity of tick hemolymph and facilitates phagocytosis. Silencing of ixo-A expression significantly decreased phagocytosis of Gram-negative bacteria such as *E. coli* and *C. indologenes* and yeast *C. albicans* (Honig Mondekova et al., 2017). Additional essential factors of hemocyte-phagocytosis are hemocyte surface receptors. Class B scavenger receptor CD36 of *H. logicornis* (HlSRB) is part of first-line host defence and plays a vital role in hemocyte-mediated phagocytosis of exogenous pathogens, such as *E. coli*. Silencing of HlSRB impeded the ability of hemocytes to phagocytise *E. coli*, resulting in a significant increase in the mortality of infected ticks (Aung et al., 2012). We can conclude, that tick hemocytes play an integral role in tick immunity and in maintaining microbial homeostasis.

In *I. scapularis* ticks colonized by *B. burgdorferi* s. l. increased expression of *pixr* gene was observed. This gene encodes a gut secreted protein with Reeler domain called PIXR (Protein of *I. scapularis* with Reeler domain). PIXR most likely takes part in immune response and its function is to limit formation and growth of bacterial biofilm. Disabling *pixr* gene results in increased biofilm in tick gut and changes in gut microbiome (Narasimhan et al., 2017). PIXR homologues can be also found in *I. ricinus* (Kotsyfakis et al., 2015). Compared to uninfected nymphs, PIXR levels were increased approximately 1.5-fold in guts of *B. burgdorferi* infected nymphs. Decreased levels of PIXR led to decline of *B. burgdorferi* population in tick gut and consequently weakened its ability to infect vertebrate hosts (mice). By increasing expression of *pixr*, *B. burgdorferi* escapes tick immune responses that might be enhanced by increased biofilm formation and variations in microbiome composition (Narasimhan et al., 2017).

### 5.7 Microbiota, blood feeding and reproduction

Tick obligate endosymbionts may play a pivotal role in ticks’ biology by affecting tick’s feeding capability, molting, reproductive fitness or development. In *H. longicornis*, *Coxiella* spp. endosymbionts play an important role in the regulation of feeding. The bacteria possess genes for production of chlorismate, a precursor of tryptophan. *Coxiella* spp. endosymbionts produced chlorismate increases biosynthesis of 5-hydroxytryptamine (serotonin) through stimulation of the expression of aromatic amino acid decarboxylase, which is necessary for catalysis decarboxylation of 5-hydroxytryptophan to serotonin. The elevated levels of serotonin in tick synganglion and midgut promotes tick feeding activity. Treating ticks with glyphosate (inhibitor of chlorismate synthesis pathway) or tetracycline (reduced abundance of *Coxiella* symbiont) notably reduced tick feeding (Hofer, 2021; Zhong et al., 2021).

*Ixodes scapularis* produces the gut protein Is86, orthologue of Bm86 of *R. microplus* or Ir86 of *I*. *ricinus*. This protein harbors EGF-like (epidermal growth factor-like) domains and is upregulated during *B. burgdorferi* s. l. infection. Levels of Is86 in the gut after feeding were 1.8-fold higher in infected ticks compared to controls. Bm86 orthologues are associated with cell growth stimulation and membrane damage restoration and are possibly involved in reshaping of tick gut during feeding. Transmission of *B. burgdorferi* from ticks feeding on mice immunized with antibodies against Is86 was significantly decreased compared to controls. These antibodies did not impact persistence of spirochetes in the tick, but likely influenced their transmission (Koči et al., 2021).

Antibiotic treatment of tick females may result in prolonged oviposition, reduction of larvae viability and weight compared to untreated ticks. Such impact on reproduction capacity observed e.g., in *A. americanum* may be explained by contraction of essential nutrients provided by endosymbionts (Zhong et al., 2007; Smith et al., 2015). Similarly, *H. longicornis* females treated with antibiotics showed a significant reduction of *Coxiella* endosymbionts in ovaries and Malpighian tubules, which resulted in prolonged feeding and oviposition and reduced oviposition (Zhang et al., 2017).

## 6 Concluding remarks

The tick microbiome is quite complex, and its composition depends on tick developmental stage and gender, geographical location, and surrounding environment. Majority of the tick microbiome is represented by transient microbial inhabitants, consisting mainly of environmental bacteria, while the number of stable tick microbiome genera is not so high. However, still most of the attention is focused on tick-borne pathogens, since they are important causative agents of human diseases. These pathogenic bacteria evolved intricate mechanisms for suppressing, evading or co-opting tick immune responses, and modifying tick behavior in a way that is beneficial for the host and pathogen. Although, they are classified as pathogens, for the tick itself they represent no danger and can contribute to its physiological maintenance together with ticks´ symbiotic bacteria. However, current knowledge of ticks´ microbiome composition and its intimate involvement in tick´s physiology, behavior and survival is still very poor and more precise microbiota member characterization is urgently needed. The involvement of the plethora of microbes in a cascade of chain reactions with a common functional outcome still needs to be untangled. Evolution of the methods of molecular biology enable more precise cell-level identification, however, there are still big gaps in the understanding of the functional mechanisms and there is high demand for the *in vitro* studies.

## Author contributions

All authors RH, MK, and KS have taken part on writing and manuscript editing and revision. RH has written, KS has planned, designed and written, and MK has designed and written, and all authors critically revised the manuscript and added ideas. All authors approved the final version of the manuscript.

## Funding

This work was supported by project VEGA 01/0404/19.

## Acknowledgments

Authors would like to acknowledge Marketa Derdakova and Juraj Koci for their support of experimental work associated and tightly connected with writing of this review.

## Conflict of interest

The authors declare that the research was conducted in the absence of any commercial or financial relationships that could be construed as a potential conflict of interest.

## Publisher’s note

All claims expressed in this article are solely those of the authors and do not necessarily represent those of their affiliated organizations, or those of the publisher, the editors and the reviewers. Any product that may be evaluated in this article, or claim that may be made by its manufacturer, is not guaranteed or endorsed by the publisher.
