# Supplementary material for: What do we know about the microbiome of I. ricinus?
Source: Front Cell Infect Microbiol. 2022 Nov 16;12:990889. doi: 10.3389/fcimb.2022.990889 (PMC9709289; doi:10.3389/fcimb.2022.990889)
Supplement: Supplementary file 1 [file Table_1.pdf]

Bacteria	Tick stage	Tick origin	Reference
Acidiphilium spp.	adults	Environmental	Rousseau et al. (2021)
Acidovorax spp.	adult females (midgut)	Environmental	Guizzo et al. (2022)
Acinetobacter spp.	adults	Environmental	Estrada-Peña et al. (2018)
	adult females (midgut)	Environmental	Guizzo et al. (2022)
	pool adults/pool nymphs	Environmental	Carpi et al. (2011)
	pool larvea and females	Breeding	Hernández-Jarguín et al. (2018)
	pool larvae	Environmental	Hamilton et al. (2021)
	nymphs	Environmental	Lejal et al. (2021)
	nymphs	Environmental	Alafaci et al. (2021)
	adults (males, females)	Environmental	Elias et al. (2021)
Acinetobacter iwoffii	adults (males, females)	Environmental	Batool et al. (2021)
Actinomyces spp.	adults	Environmental	Estrada-Peña et al. (2018)
	adults	Environmental	Carpi et al. (2011)
	pool larvae and females	Breeding	Hernández-Jarguín et al. (2018)
Actinomycetospora spp.	adults	Environmental	Estrada-Peña et al. (2018)
	adults (males, females)	Environmental	Elias et al. (2021)
Actinosynnema spp.	adults	Environmental	Carpi et al. (2011)
Aerococcus spp.	adults	Environmental	Estrada-Peña et al. (2018)
Aeromicrobium spp.	adults	Environmental	Estrada-Peña et al. (2018)
	pool adults/pool nymphs	Environmental	Carpi et al. (2011)
Aeromonas spp.	adults	Environmental	Estrada-Peña et al. (2018)
	pool adults/pool nymphs	Environmental	Carpi et al. (2011)
Afipia spp.	pool adults/pool nymphs	Environmental	Carpi et al. (2011)
Agrobacterium spp.	pool adults/pool nymphs	Environmental	Carpi et al. (2011)
Agrococcus spp.	adult females (midgut)	Environmental	Guizzo et al. (2022)
Achromobacter spp.	adult females (midgut)	Environmental	Guizzo et al. (2022)
Aliivibrio spp.	adults	Environmental	Carpi et al. (2011)
Alloprevotella spp.	adults	Environmental	Estrada-Peña et al. (2018)

Allorhizobium spp.	adults (males, females)	Environmental	Batool et al. (2021)
Amaricoccus spp.	pool adults/pool nymphs	Environmental	Carpi et al. (2011)
Amnibacterium spp.	adults	Environmental	Estrada-Peña et al. (2018)
Amycolatopsis spp.	pool larvae and females	Breeding	Hernández-Jarguín et al. (2018)
Anaerococcus spp.	adults	Environmental	Carpi et al. (2011)
Anaeromyxobacter spp.	adults	Environmental	Carpi et al. (2011)
Anaplasma spp.	adults	Environmental	Estrada-Peña et al. (2018)
	pool larvae and females	Breeding	Hernández-Jarguín et al. (2018)
	nymphs/adults	Environmental	Aivelo et al. (2019)
	nymphs	Environmental	Lejal et al. (2021)
	adults (males, females)	Environmental	Elias et al. (2021)
	adult females (midgut)	Environmental	Guizzo et al. (2022)
Anoxybacillus spp.	pool adults/pool nymphs	Environmental	Carpi et al. (2011)
	nymphs	Environmental	Alafaci et al. (2021)
Aquabacterium spp.	nymphs	Environmental	Hamilton et al. (2021)
Arcicella spp.	pool adults/pool nymphs	Environmental	Carpi et al. (2011)
Arsenophonus spp.	nymphs	Environmental	Lejal et al. (2021)
Arthrobacter spp.	adults	Environmental	Estrada-Peña et al. (2018)
	adults	Environmental	Carpi et al. (2011)
	pool adults/pool nymphs	Environmental	Carpi et al. (2011)
	pool larvae and females	Breeding	Hernández-Jarguín et al. (2018)
	adult females (midgut)	Environmental	Guizzo et al. (2022)
Aureimonas spp.	adults	Environmental	Estrada-Peña et al. (2018)
	adults (males, females)	Environmental	Elias et al. (2021)
Avibacterium spp.	adults	Environmental	Estrada-Peña et al. (2018)
Bacillus spp.	adult females (midgut)	Environmental	Guizzo et al. (2022)
	adults	Environmental	Carpi et al. (2011)
	pool larvae	Environmental	Hamilton et al. (2021)

	nymphs	Environmental	Lejal et al. (2021)
	adults (males, females)	Environmental	Elias et al. (2021)
Bacillus cereus	larvae (pools)	Environmental	Rousseau et al. (2021)
Bacillus megaterium			
Bacillus mycoides			
Bacillus pseudomycoides			
Bacillus thuringiens			
Bacillus weihenstephanensis			
Bacteorides spp.	adults	Environmental	Carpi et al. (2011)
Bartonella spp.	adults	Environmental	Estrada-Peña et al. (2018)
	adults	Environmental	Carpi et al. (2011)
Bdellovibrio spp.	adults	Environmental	Estrada-Peña et al. (2018)
	pool adults/pool nymphs	Environmental	Carpi et al. (2011)
	adult females (midgut)	Environmental	Guizzo et al. (2022)
Bibersteinia spp.	adults	Environmental	Estrada-Peña et al. (2018)
Bifidobacterium spp.	adults	Environmental	Estrada-Peña et al. (2018)
	pool larvae and females	Breeding	Hernández-Jarguín et al. (2018)
Blastococcus sp.	pool adults/pool nymphs	Environmental	Carpi et al. (2011)
Blautia sp.	adults	Environmental	Estrada-Peña et al. (2018)
Bordetella sp.	adults	Environmental	Carpi et al. (2011)
Borrelia spp.	adults	Environmental	Estrada-Peña et al. (2018)
	adults	Environmental	Carpi et al. (2011)
	pool adults/pool nymphs	Environmental	Carpi et al. (2011)
	pool larvae and females	Breeding	Hernández-Jarguín et al. (2018)
	nymphs	Environmental	Lejal et al. (2021)
	Adult females (midgut)	Environmental	Guizzo et al. (2022)
	adults (males, females)	Environmental	Elias et al. (2021)
Borrelia afzelii	nymphs/adults	Environmental	Aivelo et al. (2019)
Borrelia valaisiana			

Borrelia garinii			
Borrelia miyamotoi			
Bosea spp.	adults	Environmental	Estrada-Peña et al. (2018)
	adults (males, females)	Environmental	Elias et al. (2021)
Bradyrhizobium spp.	pool adults/pool nymphs	Environmental	Carpi et al. (2011)
	pool larvae and females	Breeding	Hernández-Jarguín et al. (2018)
	nymphs	Environmental	Hamilton et al. (2021)
Brevibacillus spp.	adults	Environmental	Estrada-Peña et al. (2018)
Brevibacterium spp.	pool adults/pool nymphs	Environmental	Carpi et al. (2011)
	pool larvae and females	Breeding	Hernández-Jarguín et al. (2018)
	pool larvae	Environmental	Hamilton et al. (2021)
	larvae (pools)	Environmental	Rousseau et al. (2021)
Brevundimonas spp.	pool adults/pool nymphs	Environmental	Carpi et al. (2011)
Brucella spp.	adults	Environmental	Carpi et al. (2011)
	pool larvae and females	Breeding	Hernández-Jarguín et al. (2018)
Burkholderia spp.	adults	Environmental	Estrada-Peña et al. (2018)
	adults	Environmental	Carpi et al. (2011)
	pool larvae	Environmental	Hamilton et al. (2021)
	nymphs	Environmental	Hamilton et al. (2021)
Campylobacter spp.	adults	Environmental	Estrada-Peña et al. (2018)
Candidatus Midichloria sp.	nymphs/adults	Environmental	Aivelo et al. (2019)
	pool larvae	Environmental	Hamilton et al. (2021)
	nymphs	Environmental	Hamilton et al. (2021)
	nymphs	Environmental	Lejal et al. (2021)
	nymphs	Environmental	Alafaci et al. (2021)
	adults (males, females)	Environmental	Elias et al. (2021)
	adults (males, females)	Environmental	Batool et al. (2021)
	adult females (midgut)	Environmental	Guizzo et al. (2022)
	pool larvae and females	Breeding	Hernández-Jarguín et al. (2018)

Candidatus Neoehrlichia sp.	adults (males, females)	Environmental	Elias et al. (2021)
	pool adults/pool nymphs	Environmental	Carpi et al. (2011)
	nymphs/adults	Environmental	Aivelo et al. (2019)
	pool larvae and females	Breeding	Hernández-Jarguín et al. (2018)
Candidatus Pelagibacter sp.	adults	Environmental	Carpi et al. (2011)
Capnocytophaga spp.	adults	Environmental	Estrada-Peña et al. (2018)
Carnobacterium spp.	adults	Environmental	Estrada-Peña et al. (2018)
Caulobacter spp.	pool adults/pool nymphs	Environmental	Carpi et al. (2011)
	pool larvae	Environmental	Hamilton et al. (2021)
	nymphs	Environmental	Hamilton et al. (2021)
Cedecae spp.	larvae (pools)	Environmental	Rousseau et al. (2021)
Cellvibrio spp.	adults	Environmental	Estrada-Peña et al. (2018)
Cloacibacterium spp.	adults	Environmental	Estrada-Peña et al. (2018)
	adult females (midgut)	Environmental	Guizzo et al. (2022)
Clostridium spp.	adults	Environmental	Carpi et al. (2011)
Clostridium baratii	larvae (pools)	Environmental	Rousseau et al. (2021)
Clostridium perfingens	larvae (pools)	Environmental	Rousseau et al. (2021)
Collulomonas	adult females (midgut)	Environmental	Guizzo et al. (2022)
Conexibacter	adults	Environmental	Carpi et al. (2011)
Congregibacter	adults	Environmental	Carpi et al. (2011)
Corynebacterium spp.	pool larvae and females	Breeding	Hernández-Jarguín et al. (2018)
	nymphs	Environmental	Alafaci et al. (2021)
	adult females (midgut)	Environmental	Guizzo et al. (2022)
Corynebacterium kroppenstedtii	larvae (pools	Environmental	Rousseau et al. (2021)
Coxiella spp.	adults	Environmental	Estrada-Peña et al. (2018)
Curtobacterium spp.	adults	Environmental	Estrada-Peña et al. (2018)
	pool adults/pool nymphs	Environmental	Carpi et al. (2011)
	adults (males, females)	Environmental	Elias et al. (2021)
	larvae (pools)	Environmental	Rousseau et al. (2021)

	adults (males, females)	Environmental	Batool et al. (2021)
Curtobacterium flaccumfaciens	larvae (pools)	Environmental	Rousseau et al. (2021)
Cutibacterium spp.	nymphs	Environmental	Alafaci et al. (2021)
	adults (males, females)	Environmental	Elias et al. (2021)
Cutibacterium acnes	larvae (pools)	Environmental	Rousseau et al. (2021)
Defluvibacter spp.	pool adults/pool nymphs	Environmental	Carpi et al. (2011)
Deinococcus spp.	adults	Environmental	Aivelo et al. (2019)
Delftia spp.	nymphs/adults	Environmental	Estrada-Peña et al. (2018)
Desemzia spp.	adults	Environmental	Estrada-Peña et al. (2018)
Desulfovibrio spp.	adults	Environmental	Guizzo et al. (2022)
	adult females (midgut)	Environmental	Estrada-Peña et al. (2018)
Devosia spp.	adults	Environmental	Carpi et al. (2011)
	pool adults/pool nymphs	Environmental	Estrada-Peña et al. (2018)
Diplorickettsia massiliensis	adults	Environmental	Mediannikov et al. (2010)
Dolosigranulum	adults	Environmental	Carpi et al. (2011)
Dorea	adults	Environmental	Estrada-Peña et al. (2018)
Duganella	adults	Environmental	Estrada-Peña et al. (2018)
Dyadobacter spp.	adults	Environmental	Carpi et al. (2011)
	pool adults/pool nymphs	Environmental	Hernández-Jarguín et al. (2018)
Ehrlichia spp.	pool larvae and females	Breeding	Elias et al. (2021)
	adults (males, females)	Environmental	Estrada-Peña et al. (2018)
Empedobacter	adults	Environmental	Carpi et al. (2011)
Enterobacter spp.	adults	Environmental	Guizzo et al. (2022)
	adult females (midgut)	Environmental	Estrada-Peña et al. (2018)
Enterococcus spp.	adults	Environmental	Carpi et al. (2011)
	pool nymphs	Environmental	Hernández-Jarguín et al. (2018)
	pool larvae and females	Breeding	Guizzo et al. (2022)
	adult females (midgut)	Environmental	Carpi et al. (2011)
Erwinia spp.	pool adults/pool nymphs	Environmental	Carpi et al. (2011)

Erythrobacter spp.	adults	Environmental	Carpi et al. (2011)
Escherichia spp.	adults	Environmental	Carpi et al. (2011)
	pool adults/pool nymphs	Environmental	Hernández-Jarguín et al. (2018)
	pool larvae and females	Breeding	Estrada-Peña et al. (2018)
Exiguobacterium spp.	adults	Environmental	Estrada-Peña et al. (2018)
Finegoldia spp.	adults	Environmental	Estrada-Peña et al. (2018)
Francisella spp.	adults	Environmental	Hernández-Jarguín et al. (2018)
	pool larvae and females	Breeding	Elias et al. (2021)
	adults (males, females)	Environmental	Carpi et al. (2011)
Frankia spp.	adults	Environmental	Estrada-Peña et al. (2018)
Frateuria spp.	adults	Environmental	Carpi et al. (2011)
Friedmanniella spp.	pool adults/pool nymphs	Environmental	Estrada-Peña et al. (2018)
Frondihabitans spp.	adults	Environmental	Estrada-Peña et al. (2018)
Fusobacterium spp.	adults	Environmental	Estrada-Peña et al. (2018)
Gemella spp.	adults	Environmental	Carpi et al. (2011)
Geobacillus spp.	adults	Environmental	Carpi et al. (2011)
Geobacter spp.	adults	Environmental	Elias et al. (2021)
Geodermatophilus spp.	adults (males, females)	Environmental	Hernández-Jarguín et al. (2018)
Gilliamella spp.	pool larvae and females	Breeding	Estrada-Peña et al. (2018)
Granulicatella spp.	adults	Environmental	Carpi et al. (2011)
Haliscomenobacter spp.	pool adults/pool nymphs	Environmental	Carpi et al. (2011)
Halogeometricum spp.	adults	Environmental	Elias et al. (2021)
Halomonas spp.	adults (males, females)	Environmental	Batool et al. (2021)
	adults (males, females)	Environmental	Carpi et al. (2011)
Halorubrum spp.	adults	Environmental	Carpi et al. (2011)
Heliobacterium spp.	adults	Environmental	Guizzo et al. (2022)
Herbiconiux spp.	adult females (midgut)	Environmental	Guizzo et al. (2022)
Hydrogenophaga spp.	adult females (midgut)	Environmental	Carpi et al. (2011)
Hymenobacter spp.	pool adults/pool nymphs	Environmental	Elias et al. (2021)

	adults (males, females)	Environmental	Carpi et al. (2011)
Hyphomicrobium spp.	pool adults/pool nymphs	Environmental	Carpi et al. (2011)
Chlorobium spp.	adults	Environmental	Guizzo et al. (2022)
Chryseobacterium spp.	adult females (midgut)	Environmental	Lejal et al. (2021)
	nymphs	Environmental	Guizzo et al. (2022)
Ideonella spp.	adult females (midgut)	Environmental	Guizzo et al. (2022)
Ignatzschineria spp.	adult females (midgut)	Environmental	Estrada-Peña et al. (2018)
Janibacter spp.	adults	Environmental	Estrada-Peña et al. (2018)
Jatrophihabitans spp.	adults	Environmental	Estrada-Peña et al. (2018)
Jeotgalicoccus spp.	adults	Environmental	Estrada-Peña et al. (2018)
Kineococcus spp.	adults	Environmental	Carpi et al. (2011)
	adults	Environmental	Carpi et al. (2011)
	pool adults/pool nymphs	Environmental	Elias et al. (2021)
	adults (males, females)	Environmental	Guizzo et al. (2022)
	adult females (midgut)	Environmental	Carpi et al. (2011)
Kineosporia spp.	pool adults/pool nymphs	Environmental	Carpi et al. (2011)
Kingella spp.	adults	Environmental	Hernández-Jarguín et al. (2018)
Klebsiella spp.	pool larvae and females	Breeding	Estrada-Peña et al. (2018)
Knoellia spp.	adults	Environmental	Estrada-Peña et al. (2018)
Kocuria spp.	adults	Environmental	Hernández-Jarguín et al. (2018)
Kurthia spp.	pool larvae and females	Breeding	Carpi et al. (2011)
Lactobacillus spp.	adults	Environmental	Estrada-Peña et al. (2018)
Lactococcus spp.	adults	Environmental	Aivelo et al. (2019)
Lariskella spp.	nymphs/adults	Environmental	Carpi et al. (2011)
Leadbetterella spp.	pool adults/pool nymphs	Environmental	Guizzo et al. (2022)
Legionella spp.	adult females (midgut)	Environmental	Carpi et al. (2011)
Leifsonia spp.	adults	Environmental	Guizzo et al. (2022)
	adult females (midgut)	Environmental	Hernández-Jarguín et al. (2018)
Leptotrichia spp.	pool larvae and females	Breeding	Estrada-Peña et al. (2018)

Leuconostoc spp.	adults	Environmental	Guizzo et al. (2022)
Limnobacter spp.	adult females (midgut)	Environmental	Guizzo et al. (2022)
Limnohabitans spp.	adult females (midgut)	Environmental	Carpi et al. (2011)
Listeria	adults	Environmental	Estrada-Peña et al. (2018)
Luteibacter	adults	Environmental	Hamilton et al. (2021)
	pool larvae	Environmental	Estrada-Peña et al. (2018)
Luteolibacter	adults	Environmental	Rousseau et al. (2021)
Lysinibacillus fusiformis	larvae (pools)	Environmental	Guizzo et al. (2022)
Lysinibacillus spp.	adult females (midgut)	Environmental	Estrada-Peña et al. (2018)
Lysobacter spp.	adults	Environmental	Carpi et al. (2011)
	adults	Environmental	Hernández-Jarguín et al. (2018)
	pool larvae and females	Breeding	Estrada-Peña et al. (2018)
Macrococcus spp.	adults	Environmental	Carpi et al. (2011)
Magnetospirillum spp.	adults	Environmental	Carpi et al. (2011)
	pool adults/pool nymphs	Environmental	Estrada-Peña et al. (2018)
Marmoricola spp.	adults	Environmental	Rousseau et al. (2021)
Massilia timonae	larvae (pools)	Environmental	Hernández-Jarguín et al. (2018)
Mesorhizobium spp.	pool larvae and females	Breeding	Hamilton et al. (2021)
	pool larvae	Environmental	Hamilton et al. (2021)
	nymphs	Environmental	Hamilton et al. (2021)
	nymphs	Environmental	Carpi et al. (2011)
Methylacidiphilum spp.	adults	Environmental	Carpi et al. (2011)
Methylobacterium spp.	pool adults/pool nymphs	Environmental	Hamilton et al. (2021)
	pool larvae	Environmental	Hamilton et al. (2021)
	nymphs	Environmental	Lejal et al. (2021)
	nymphs	Environmental	Alafaci et al. (2021)
	nymphs	Environmental	Elias et al. (2021)
	adults (males, females)	Environmental	Batool et al. (2021)
	adults (males, females)	Environmental	Guizzo et al. (2022)

	adult females (midgut)	Environmental	Carpi et al. (2011)
Methylopila spp.	pool adults/pool nymphs	Environmental	Guizzo et al. (2022)
Microbacterium spp.	adult females (midgut)	Environmental	Estrada-Peña et al. (2018)
Micrococcus spp.	adults	Environmental	Carpi et al. (2011)
	pool adults/pool nymphs	Environmental	Hamilton et al. (2021)
	pool larvae	Environmental	Guizzo et al. (2022)
	adult females (midgut)	Environmental	Rousseau et al. (2021)
Micrococcus luteus	larvae (pools)	Environmental	Carpi et al. (2011)
Micromonospora spp.	adults	Environmental	Carpi et al. (2011)
Moritella spp.	adults	Environmental	Hernández-Jarguín et al. (2018)
Mucilaginibacter spp.	pool larvae and females	Breeding	Estrada-Peña et al. (2018)
Mycobacterium	adults	Environmental	Carpi et al. (2011)
		Environmental	Carpi et al. (2011)
	pool adults/pool nymphs	Environmental	Hamilton et al. (2021)
	nymphs	Environmental	Lejal et al. (2021)
		Environmental	Alafaci et al. (2021)
		Environmental	Elias et al. (2021)
	adults (males, females)	Environmental	Batool et al. (2021)
		Environmental	Guizzo et al. (2022)
	adult females (midgut)	Environmental	Guizzo et al. (2022)
Mycobateroides spp.	adult females (midgut)	Environmental	Guizzo et al. (2022)
Mycolicibacterium spp.	adult females (midgut)	Environmental	Carpi et al. (2011)
Myxococcus spp.	adults	Environmental	Hamilton et al. (2021)
	nymphs	Environmental	Estrada-Peña et al. (2018)
Nakamurella spp.	adults	Environmental	Carpi et al. (2011)
	adults	Environmental	Elias et al. (2021)
	adults (males, females)	Environmental	Estrada-Peña et al. (2018)
Neoehrlichia spp.	adults	Environmental	Batool et al. (2021)
Neorhizobium spp.	adults (males, females)	Environmental	Hernández-Jarguín et al. (2018)

Neorickettsia spp.	pool larvae and females	Breeding	Carpi et al. (2011)
Niastella spp.	pool adults/pool nymphs	Environmental	Carpi et al. (2011)
Nocardioides spp.	pool adults/pool nymphs	Environmental	Carpi et al. (2011)
Nostoc spp.	adults	Environmental	Carpi et al. (2011)
Novosphingobium spp.	pool adults/pool nymphs	Environmental	Carpi et al. (2011)
Oceanicola spp.	adults	Environmental	Carpi et al. (2011)
Octadecabacter spp.	adults	Environmental	Estrada-Peña et al. (2018)
Odoribacter spp.	adults	Environmental	Carpi et al. (2011)
Ochrobacterum spp.	pool adults/pool nymphs	Environmental	Guizzo et al. (2022)
Pantibacter spp.	adult females (midgut)	Environmental	Guizzo et al. (2022)
Pantoea spp.	adult females (midgut)	Environmental	Alafaci et al. (2021)
Paracoccus spp.	nymphs	Environmental	Batool et al. (2021)
Pararhizobium spp.	adults (males, females)	Environmental	Estrada-Peña et al. (2018)
Patulibacterspp.	adult	Environmental	Carpi et al. (2011)
	pool adults/pool nymphs	Environmental	Rousseau et al. (2021)
Peanibacillus amylaticus	larvae (pools)	Environmental	Rousseau et al. (2021)
Peanibacillus pabuli	larvae (pools)	Environmental	Rousseau et al. (2021)
Peanibacillus taiwanensis	larvae (pools)	Environmental	Estrada-Peña et al. (2018)
Pedobacter spp.	adults	Environmental	Carpi et al. (2011)
	pool adults/pool nymphs	Environmental	Guizzo et al. (2022)
Pelomonas spp.	adult females (midgut)	Environmental	Guizzo et al. (2022)
	adult females (midgut)	Environmental	Estrada-Peña et al. (2018)
Phenylobacterium spp.	adults	Environmental	Estrada-Peña et al. (2018)
Phycicoccus spp.	adults	Environmental	Estrada-Peña et al. (2018)
Phyllobacterium spp.	adults	Environmental	Carpi et al. (2011)
Pleomorhomonas spp.	pool adults/pool nymphs	Environmental	Carpi et al. (2011)
Polaromonas spp.	adults	Environmental	Estrada-Peña et al. (2018)
Prevotella spp.	adults	Environmental	Carpi et al. (2011)
	adults	Environmental	Estrada-Peña et al. (2018)

Propionibacterium spp.	adults	Environmental	Estrada-Peña et al. (2018)
	pool adults/pool nymphs	Environmental	Carpi et al. (2011)
	pool larvae and females	Breeding	Hernández-Jarguín et al. (2018)
Providencia spp.	adults	Environmental	Estrada-Peña et al. (2018)
	pool adults/pool nymphs	Environmental	Carpi et al. (2011)
Pseonocardia spp.	pool adults/pool nymphs	Environmental	Carpi et al. (2011)
Pseudarcicella spp.	adults	Environmental	Estrada-Peña et al. (2018)
Pseudoalteromonas spp.	adults	Environmental	Carpi et al. (2011)
Pseudoclavibacter spp.	adult females (midgut)	Environmental	Guizzo et al. (2022)
Pseudomonas spp.	pool adults/pool nymphs	Environmental	Carpi et al. (2011)
	pool larvae and females	Breeding	Hernández-Jarguín et al. (2018)
	nymphs/adults	Environmental	Aivelo et al. (2019)
	pool larvae	Environmental	Hamilton et al. (2021)
	nymphs	Environmental	Hamilton et al. (2021)
		Environmental	Lejal et al. (2021)
	adults (males, females)	Environmental	Elias et al. (2021)
		Environmental	Batool et al. (2021)
	adult females (midgut)	Environmental	Guizzo et al. (2022)
Pseudorhodobacter spp.	adult females (midgut)	Environmental	Guizzo et al. (2022)
Pseudoxanthomonas spp.	adults	Environmental	Estrada-Peña et al. (2018)
	pool nymph	Environmental	Carpi et al. (2011)
Psychrobacter spp.	nymphs	Environmental	Alafaci et al. (2021)
Pyrococcus spp.	adults	Environmental	Carpi et al. (2011)
Pyschroflexus spp.	adults	Environmental	Carpi et al. (2011)
Quadrisphaera spp.	pool adults/pool nymphs	Environmental	Carpi et al. (2011)
Rahnella spp.	pool larvae	Environmental	Hamilton et al. (2021)
Ralstonia spp.	adults	Environmental	Carpi et al. (2011)
	pool larvae	Environmental	Hamilton et al. (2021)
	nymphs	Environmental	Hamilton et al. (2021)

		Environmental	Alafaci et al. (2021)
	adult females (midgut)	Environmental	Guizzo et al. (2022)
Raoultella spp.	pool larvae	Environmental	Hamilton et al. (2021)
Reinekea spp.	adults	Environmental	Carpi et al. (2011)
Renibacterium spp.	adults	Environmental	Carpi et al. (2011)
Resomonas spp.	pool adults/pool nymphs	Environmental	Carpi et al. (2011)
Rhizobium spp.	pool adults/pool nymphs	Environmental	Carpi et al. (2011)
	pool larvae	Environmental	Hamilton et al. (2021)
	nymphs	Environmental	Hamilton et al. (2021)
		Environmental	Lejal et al. (2021)
	adults (males, females)	Environmental	Batool et al. (2021)
	adult females (midgut)	Environmental	Guizzo et al. (2022)
Rhodanobacter spp.	adults	Environmental	Estrada-Peña et al. (2018)
Rhodococcus spp.	adults	Environmental	Carpi et al. (2011)
	pool larvae and females	Breeding	Hernández-Jarguín et al. (2018)
	nymphs	Environmental	Alafaci et al. (2021)
	adults (males, females)	Environmental	Elias et al. (2021)
	adult females (midgut)	Environmental	Guizzo et al. (2022)
Rhodomicrobium spp.	adults	Environmental	Carpi et al. (2011)
Rhodopseudomonas spp.	adults	Environmental	Estrada-Peña et al. (2018)
	adults	Environmental	Carpi et al. (2011)
	pool larvae	Environmental	Hamilton et al. (2021)
	nymphs	Environmental	Hamilton et al. (2021)
Rhodotobacter spp.	adults	Environmental	Carpi et al. (2011)
Rickettsia spp.	adults	Environmental	Estrada-Peña et al. (2018)
	adult s	Environmental	Carpi et al. (2011)
	pool adults/pool nymphs	Environmental	Carpi et al. (2011)
	pool larvae and females	Breeding	Hernández-Jarguín et al. (2018)
	nymphs	Environmental	Lejal et al. (2021)

	nymphs	Environmental	Alafaci et al. (2021)
	adults (males, females)	Environmental	Elias et al. (2021)
		Environmental	Batool et al. (2021)
	adult females (midgut)	Environmental	Guizzo et al. (2022)
Rickettsia helvetica	nymphs/adults	Environmental	Aivelo et al. (2019)
Rickettsia monacensis			
Rickettsiella spp.	pool adults/pool nymphs	Environmental	Carpi et al. (2011)
	adults	Environmental	Estrada-Peña et al. (2018)
	pool larvae and females	Breeding	Hernández-Jarguín et al. (2018)
	nymphs/adults	Environmental	Aivelo et al. (2019)
	nymphs	Environmental	Lejal et al. (2021)
		Environmental	Alafaci et al. (2021)
	adult females (midgut)	Environmental	Guizzo et al. (2022)
Romboutsia spp.	adult females (midgut)	Environmental	Guizzo et al. (2022)
Roseburia spp.	adults	Environmental	Hernández-Jarguín et al. (2018)
Roseomonas spp.	adults	Environmental	Hernández-Jarguín et al. (2018)
Rothia amarae spp.	larvae (pools)	Environmental	Rousseau et al. (2021)
Rothia spp.	adult females (midgut)	Environmental	Guizzo et al. (2022)
Ruminococcus spp.	adults	Environmental	Hernández-Jarguín et al. (2018)
Salmonella spp.	adults	Environmental	Carpi et al. (2011)
Segetibacter spp.	adults	Environmental	Hernández-Jarguín et al. (2018)
Serratia spp.	adult females (midgut)	Environmental	Guizzo et al. (2022)
Schewanella spp.	adults	Environmental	Carpi et al. (2011)
Sorangium spp.	adults	Environmental	Hernández-Jarguín et al. (2018)
	adults	Environmental	Carpi et al. (2011)
	pool adults/pool nymphs	Environmental	Carpi et al. (2011)
Sphingobacterium spp.	adults	Environmental	Hernández-Jarguín et al. (2018)
	pool adults/pool nymphs	Environmental	Carpi et al. (2011)
	nymphs	Environmental	Hamilton et al. (2021)

Sphingobium spp.	pool adults/pool nymphs	Environmental	Carpi et al. (2011)
	pool larvae	Environmental	Hamilton et al. (2021)
	nymphs	Environmental	Hamilton et al. (2021)
Sphingomonas spp.	pool adults/pool nymphs	Environmental	Carpi et al. (2011)
	pool larvae and females	Breeding	Hernández-Jarguín et al. (2018)
	nymphs/adults	Environmental	Aivelo et al. (2019)
	pool larvae	Environmental	Hamilton et al. (2021)
	nymphs	Environmental	Alafaci et al. (2021)
	adults (males, females)	Environmental	Elias et al. (2021)
	adults (males, females)	Environmental	Batool et al. (2021)
	adult females (midgut)	Environmental	Guizzo et al. (2022)
Sphingopyxis spp.	adults	Environmental	Carpi et al. (2011)
Sphingorhabdus spp.	nymphs	Environmental	Hamilton et al. (2021)
Sphingotrophomonas spp.	nymphs	Environmental	Hamilton et al. (2021)
Spinghopyxis spp.	pool adults/pool nymphs	Environmental	Carpi et al. (2011)
Spiroplasma spp.	adult	Environmental	Estrada-Peña et al. (2018)
	pool larvae and females	Breeding	Hernández-Jarguín et al. (2018)
	nymphs/adults	Environmental	Aivelo et al. (2019)
	pool larvae	Environmental	Hamilton et al. (2021)
	nymphs	Environmental	Hamilton et al. (2021)
	nymphs	Environmental	Lejal et al. (2021)
	nymphs	Environmental	Alafaci et al. (2021)
	adults (males, females)	Environmental	Elias et al. (2021)
	adult females (midgut)	Environmental	Guizzo et al. (2022)
Spirosoma spp.	adults	Environmental	Hernández-Jarguín et al. (2018)
	pool adults/pool nymphs	Environmental	Carpi et al. (2011)
	adults (males, females)	Environmental	Elias et al. (2021)
Staphylococcus spp.	pool adults/pool nymphs	Environmental	Carpi et al. (2011)
	pool larvae and females	Breeding	Hernández-Jarguín et al. (2018)

	nymphs	Environmental	Alafaci et al. (2021)
	adults (males, females)	Environmental	Elias et al. (2021)
	larvae (pools)	Environmental	Rousseau et al. (2021)
	adult females (midgut)	Environmental	Guizzo et al. (2022)
Staphylococcus epidermidis	larvae (pools)	Environmental	Rousseau et al. (2021)
Staphylococcus hominis			
Staphylococcus xylosus			
Staphylococcus capitis			
Stenotrophomonas spp.	adults	Environmental	Estrada-Peña et al. (2018)
	pool adults/pool nymphs	Environmental	Carpi et al. (2011)
	pool larvae	Environmental	Hamilton et al. (2021)
	nymphs	Environmental	Hamilton et al. (2021)
		Environmental	Lejal et al. (2021)
	adult females (midgut)	Environmental	Guizzo et al. (2022)
Streptococcus spp.	pool nymphs	Environmental	Carpi et al. (2011)
	pool larvae and females	Breeding	Hernández-Jarguín et al. (2018)
	adult females (midgut)	Environmental	Guizzo et al. (2022)
Streptomyces spp.	adults	Environmental	Carpi et al. (2011)
	pool adults/pool nymphs	Environmental	Carpi et al. (2011)
	adult females (midgut)	Environmental	Guizzo et al. (2022)
Syntrophus spp.	adults	Environmental	Carpi et al. (2011)
Termotoga spp.	adults	Environmental	Carpi et al. (2011)
Terriglobus spp.	adults	Environmental	Estrada-Peña et al. (2018)
Terrimonas spp.	pool adults/pool nymphs	Environmental	Carpi et al. (2011)
Thauera spp.	adults	Environmental	Carpi et al. (2011)
Thermanaerovibrio spp.	adults	Environmental	Carpi et al. (2011)
Thiothrix spp.	adult females (midgut)	Environmental	Guizzo et al. (2022)
Thorsellia spp.	adult females (midgut)	Environmental	Guizzo et al. (2022)
Treponema spp.	pool larvae and females	Breeding	Hernández-Jarguín et al. (2018)

Tulomonas spp.	adults	Environmental	Carpi et al. (2011)
Variovorax spp.	pool larvae and females	Breeding	Hernández-Jarguín et al. (2018)
	pool larvae	Environmental	Hamilton et al. (2021)
	nymphs	Environmental	Hamilton et al. (2021)
Veillonella spp.	adults	Environmental	Estrada-Peña et al. (2018)
	adult females (midgut)	Environmental	Guizzo et al. (2022)
Verrucomirobium spp.	adults	Environmental	Carpi et al. (2011)
Vibrio spp.	adults	Environmental	Carpi et al. (2011)
Wautersiella spp.	adults	Environmental	Estrada-Peña et al. (2018)
Weissella spp.	adults	Environmental	Estrada-Peña et al. (2018)
Williamsia spp.	adults	Environmental	Estrada-Peña et al. (2018)
	nymphs	Environmental	Lejal et al. (2021)
	adult females (midgut)	Environmental	Guizzo et al. (2022)
Wolbachia spp.	pool nymps	Environmental	Carpi et al. (2011)
	pool larvae and females	Breeding	Hernández-Jarguín et al. (2018)
	nymphs	Environmental	Lejal et al. (2021)
	nymphs	Environmental	Alafaci et al. (2021)
	adult females (midgut)	Environmental	Guizzo et al. (2022)
Xantomonas spp.	adults	Environmental	Carpi et al. (2011)
	pool nymphs	Environmental	Carpi et al. (2011)
Xylella spp.	adults	Environmental	Carpi et al. (2011)

Supplementary Table 1. Bacteria detected in *Ixodes ricinus.*

References

Aivelo, T., Norberg, A., and Tschirren, B. (2019). Bacterial microbiota composition of *Ixodes ricinus* ticks: the role of environmental variation, tick characteristics and microbial interactions. *PeerJ* 7, e8217. doi: 10.7717/peerj.8217

Alafaci, A., Crépin, A., Beaubert, S., Berjeaud, J. M., Delafont, V., and Verdon, J. (2021). Exploring the individual bacterial microbiota of questing *Ixodes ricinus* nymphs. *Microorganisms* 9 (7), 1526. doi: 10.3390/microorganisms9071526

Batool, M., Blazier, J. C., Rogovska, Y. V., Wang, J., Liu, S., Nebogatkin, I. V., et al. (2021). Metagenomic analysis of individually analyzed ticks from Eastern Europe demonstrates regional and sex-dependent differences in the microbiota of *Ixodes ricinus*. *Ticks Tick Borne Dis*. 12 (5), 101768. doi: 10.1016/ j.ttbdis.2021.101768

Carpi, G., Cagnacci, F., Wittekindt, N. E., Zhao, F., Qi, J., Tomsho, L. P., et al. (2011). Metagenomic profile of the bacterial communities associated with *Ixodes ricinus* ticks. *PLoS One* 6 (10), e25604. doi: 10.1371/journal.pone.0025604

Elias, L., Hearn, A.-J. M., Blazier, J. C., Rogovska, Y. V., Wang, J., Li, S., et al. (2021). The microbiota of *Ixodes ricinus* and *Dermacentor reticulatus* ticks collected from a highly populated city of Eastern Europe. *Microb. Ecol*. doi: 10.1007/s00248-021-01921-6

Estrada-Peña, A., Cabezas-Cruz, A., Pollet, T., Vayssier-Taussat, M., and Cosson, J. F. (2018). High throughput sequencing and network analysis disentangle the microbial communities of ticks and hosts within and between ecosystems. *Front. Cell Infect. Microbiol*. 8, 236. doi: 10.3389/fcimb.2018.00236

Guizzo, M. G., Dolezelikova, K., Neupane, S., Frantova, H., Hrbatova, A., Pafco, B., et al. (2022). Characterization and manipulation of the bacterial community in the midgut of *Ixodes ricinus*. *Parasit. Vectors* 15 (1), 248. doi: 10.1186/s13071-022-05362-z

Hamilton, P. T., Maluenda, E., Sarr, A., Belli, A., Hurry, G., Duron, O., et al. (2021). *Borrelia afzelii* infection in the rodent host has dramatic effects on the bacterial microbiome of *Ixodes ricinus* ticks. *Appl. Environ. Microbiol*. 87 (18), e0064121. doi: 10.1128/aem.00641-21

Hernández-Jarguín, A., Díaz-Sánchez, S., Villar, M., and de la Fuente, J. (2018). Integrated metatranscriptomics and metaproteomics for the characterization of bacterial microbiota in unfed *Ixodes ricinus. Ticks Tick Borne Dis*. 9 (5), 1241–1251. doi: 10.1016/j.ttbdis.2018.04.020

Lejal, E., Chiquet, J., Aubert, J., Robin, S., Estrada-Peña, A., Rue, O., et al. (2021). Temporal patterns in *Ixodes ricinus* microbial communities: an insight into tick-borne microbe interactions. *Microbiome* 9 (1), 153. doi: 10.1186/s40168-021-01051-8

Mediannikov, O., Sekeyová, Z., Birg, M. L., and Raoult, D. (2010). A novel obligate intracellular gamma-proteobacterium associated with ixodid ticks, *Diplorickettsia massiliensis*, gen. nov., sp. nov. *PLoS One* 5 (7), e11478. doi: 10.1371/journal.pone.0011478

Rousseau, R., Vanwambeke, S. O., Boland, C., and Mori, M. (2021). The isolation of culturable bacteria in *Ixodes ricinus* ticks of a Belgian peri-urban forest uncovers opportunistic bacteria potentially important for public health. *Int. J. Environ. Res. Publ. Health* 18 (22), 12134. doi: 10.3390/ijerph182212134
