# Supplementary material for: What do we know about the microbiome of I. ricinus?
Source: Front Cell Infect Microbiol. 2022 Nov 16;12:990889. doi: 10.3389/fcimb.2022.990889 (PMC9709289; doi:10.3389/fcimb.2022.990889)
Supplement: Supplementary file 2 [file Table_2.pdf]

Virus	Virus family	Tick stage	Tick origin	Reference
Ixodes ricinus orinovirus-like virus 1	*Nyamiviridae*	N/A (mixed pool)	Environmental	Sameroff et al. (2022)
Groutenhout norwavirus (L/S)	*Orthonairoviridae*			
Bronnoya virus (L/M)	*Peribunyaviridae*			
Ixodes ricinus bunyavirus-like virus 1 (L/M)				
Ixodes ricinus picorna-like virus 1	*Unclassified*			
Ixodes ricinus sobemo-like virus 1				
Ixodes ricinus noda-like virus 1				
Bronnoya virus	*Bunyaviridae*	pool (nymphs), pool adults	Environmental	Petterson et al. (2017)
Norway nairovirus 1				
Norway phlebovirus 1				
Norway luteo-like virus 1	*Luteoviridae*			
Norway luteo-like virus 2				
Norway luteo-like virus 3				
Norway luteo-like virus 4				
Norway mononegavirus 1	*Mononegavirales*			
Norway partiti-like virus 1	*Partitiviridae*			
Coltivirus	*Reoviridae*	adults, pooled nymphs	Environmental	Moutailler et al. (2016a)
Hantavirus	*Bunyaviridae*			
Nairovirus				
Orthobunyavirus				
Phlebovirus				
Tospovirus				
Lyssavirus	*Rhabdoviridae*			
Novirhabdovirus				
Cytorhabdovirus				
Ephemerovirus				
Nucleorhabdovirus				
Lyssavirus				

Sigmavirus				
Flavivirus				
Pestivirus	*Flaviviridae*			
Hepacivirus				
Hepatitis GB				
Alphavirus	*Togaviridae*			
Influenzavirus A	*Orthomyxoviridae*			
Sulina virus	*Orthonairoviridae*	N/A	Environmental	Tomazatos et al. (2021)
Grotenhout virus S/L	*Bunyaviridae*	pools (adults, nymphs, larvae)	Environmental	Vanmechelen et al. (2021)
Leuven phlebovirus virus S/L				
Chimay rhabdovirus	*Rhabdoviridae*			
-	*Botourmiaviridae*			
-	*Chrysoviridae*			
-	*Endornaviridae*			
-	*Hypoviridae*			
-	*Polymycoviridae*			
-	*Quadriviridae*			
Uukuniemi phlebovirus	*(Phenuiviridae)*	N/A	Environmental	Papa et al. (2018)
Kemerovo virus complex *(Tribeč, Lipovnik)*	*Reoviridae*	N/A	Environmental	Grešíková (1972)
Tick-borne encephalitis virus	*Flaviviridae*	pools (adults), pools (nymphs)<br>pools (larvae)	Environmental	*Vikse et al. (2020); Ott et al. (2020); Petersen et al. (2019)
Louping-ill virus	*Flaviviridae*	N/A	Environmental	Gaunt et al. (1997)
Eyach virus	*Reoviridae*	N/A	Environmental	Rehse-Kupper et al. (1976)
Crimean-Congo hemorrhagic fever virus	*Bunyaviridae*	N/A	Environmental	Gergova et al. (2012); Sultankulova et al. (2022)
		adults		Sherifi et al. (2014)

Alongshan virus	*Flaviviridae*	N/A	Environmental	Kuivannen et al. (2019)
Jimgmen-like virus				
Murid herpesvirus 4	*Herpesviridae*	larvae, nymphs	Environmental	Ficová et al. (2011)

Supplementary Table 2. Viruses detected in *Ixodes ricinus.*

** For Tick borne encephalitis virus the number of citations is very high, therefore only a few are mentioned as examples.*

References

Ficová, M., Betáková, T., Pančik, P., Václav, R., Prokop, P., Halásová, Z., et al. (2011). Molecular detection of murine herpesvirus 68 in ticks feeding on free-living reptiles. *Microb. Ecol.* 62 (4), 862–867. doi: 10.1007/s00248-011-9907-7

Gaunt, M. W., Jones, L. D., Laurenson, K., Hudson, P. J., Reid, H. W., and Gould, E. A. (1997). Definitive identification of louping ill virus by RT-PCR and sequencing in field populations of *Ixodes ricinus* on the Lochindorb Estate. *Arch. Virol.* 142 (6), 1181–1191. doi: 10.1007/s007050050151

Gergova, I., Kunchev, M., and Kamarinchev, B. (2012). Crimean-Congo hemorrhagic fever virus-tick survey in endemic areas in Bulgaria. *J. Med. Virol.* 84 (4), 608–614. doi: 10.1002/jmv.23214

Grešíková, M. (1972). Studies on tick-borne arboviruses isolated in central Europe. Biologické Práce 18 (2), 1–116.

Kuivanen, S., Levanov, L., Kareinen, L., Sironen, T., Jääskeläinen, A. J., Plyusnin, I., et al. (2019). Detection of novel tick-borne pathogen, Alongshan virus, in *Ixodes ricinus* ticks, south-eastern Finland. *Euro Surveill* 24 (27), pii=1900394. doi: 10.2807/1560-7917.es.2019.24.27.1900394

Moutailler, S., Popovici, I., Devillers, E., Vayssier-Taussat, M., and Eloit, M. (2016a). Diversity of viruses in *Ixodes ricinus*, and characterization of a neurotropic strain of Eyach virus. *New Microbes New Infect*. 11, 71–81. doi: 10.1016/j.nmni.2016.02.012

Ott, D., Ulrich, K., Ginsbach, P., Öhme, R., Bock-Hensley, O., Falk, U., et al. (2020). Tick-borne encephalitis virus (TBEV) prevalence in field-collected ticks (*Ixodes ricinus*) and phylogenetic, structural and virulence analysis in a TBE highrisk endemic area in southwestern Germany. *Parasit. Vectors* 13 (1), 303. doi: 10.1186/s13071-020-04146-7

Papa, A., Zelená, H., Papadopoulou, E., and Mrázek, J. (2018). Uukuniemi virus, Czech republic. *Ticks Tick Borne Dis*. 9 (5), 1129–1132. doi: 10.1016/j.ttbdis.2018.04.011

Petersen, A., Rosenstierne, M. W., Rasmussen, M., Fuursted, K., Nielsen, H. V., O'Brien Andersen, L., et al. (2019). Field samplings of *Ixodes ricinus* ticks from a tick-borne encephalitis virus micro-focus in northern Zealand, Denmark. *Ticks Tick Borne Dis*. 10 (5), 1028–1032. doi: 10.1016/j.ttbdis.2019.05.005

Pettersson, J. H., Shi, M., Bohlin, J., Eldholm, V., Brynildsrud, O. B., Paulsen, K. M., et al. (2017). Characterizing the virome of *Ixodes ricinus* ticks from northern Europe. *Sci. Rep.* 7 (1), 10870. doi: 10.1038/s41598-017-11439-y

Sameroff, S., Tokarz, R., Vucelja, M., Jain, K., Oleynik, A., Boljfetić, M., et al. (2022). Virome of *Ixodes ricinus, Dermacentor reticulatus*, and *Haemaphysalis concinna* ticks from Croatia. *Viruses* 14 (5), 929. doi: 10.3390/v14050929

Sherifi, K., Cadar, D., Muji, S., Robaj, A., Ahmeti, S., Jakupi, X., et al. (2014). Crimean-Congo hemorrhagic fever virus clades V and VI (Europe 1 and 2) in ticks in Kosovo. *PLoS Negl. Trop. Dis.* 8 (9), e3168. doi: 10.1371/journal.pntd.0003168

Sultankulova, K. T., Shynybekova, G. O., Kozhabergenov, N. S., Mukhami, N. N., Chervyakova, O. V., Burashev, Y. D., et al. (2022). The prevalence and genetic variants of the CCHF virus circulating among ticks in the southern regions of Kazakhstan. *Pathogens* 11 (8), 841. doi: 10.3390/pathogens11080841

Tomazatos, A., von Possel, R., Pekarek, N., Holm, T., Rieger, T., Baum, H., et al. (2021). Discovery and genetic characterization of a novel orthonairovirus in *Ixodes ricinus* ticks from Danube delta. *Infect. Genet. Evol*. 88, 104704. doi: 10.1016/j.meegid.2021.104704

Vanmechelen, B., Merino, M., Vergote, V., Laenen, L., Thijssen, M., Martί-Carreras, J., et al. (2021). Exploration of the *Ixodes ricinus* virosphere unveils an extensive virus diversity including novel coltiviruses and other reoviruses. *Virus Evol*. 7 (2), veab066. doi: 10.1093/ve/veab066

Vikse, R., Paulsen, K. M., Edgar, K. S., Pettersson, J., Ottesen, P. S., Okbaldet, Y. B., et al. (2020). Geographical distribution and prevalence of tick-borne encephalitis virus in questing *Ixodes ricinus* ticks and phylogeographic structure of the *Ixodes ricinus* vector in Norway. *Zoonoses Public Health* 67 (4), 370–381. doi: 10.1111/zph.12696
