# Supplementary material for: What do we know about the microbiome of I. ricinus?
Source: Front Cell Infect Microbiol. 2022 Nov 16;12:990889. doi: 10.3389/fcimb.2022.990889 (PMC9709289; doi:10.3389/fcimb.2022.990889)
Supplement: Supplementary file 3 [file Table_3.pdf]

Organism	Tick stage	Tick origin	Reference
**Apicomplexa**			
Babesia venatorum	nymphs	Environmental	Nebbak et al. (2019)
Babesia clade I			
Babesia clade X	adults, nymphs	Environmental	Azagi et al. (2021)
Babesia microti			
Babesia venatorum			
Babesia capreoli	adults, nymphs, larvae	Environmental (from birds)	Wilhelmsson et al. (2021)
Babesia canis	adults, nymphs, larvae	Environmental/Urban	Liberska et al. (2021)
Babesia microti			
Babesia venatorum			
Babesia divergens	adults, nymphs, larvae	Environmental	Karlsson et al. (2015)
Babesia vulpes	adults from red foxes	Environmental	Checa et al. (2018)
Theileria capreoli	larvae, nymphs, adults feeding on cervids	Environmental	Hamšíková et al. (2016a)
Theileria sp. OT3	adults feeding on cervids	Environmental	Fernández et al. (2022)
Hepatozoon canis	adults	Environmental	Hamšíková et al. (2016b)
Toxoplasma gondii	N/A	Environmental	Adamska and Skotarczak (2017)
**Kinetoplastea**			
Trypanosoma sp. Bratislava1	adults	Environmental	Luu et al. (2020)
**Microsporidia**			
Nosema slovaca	N/A	Environmental	Weiser and Rehácek (1975)
Unikaryon (Nosema) ixodis	N/A	Environmental	Weiser et al. (1975)
**Fungi**			
Verticillium lecanii			
Paecilomyces farinosus			
Beauveria bassiana			
Beauveria brongniartii			
Paecilomyces fumosoroseus			
Verticillium aranearum	nymphs	Environmental	Kalsbeek et al. (1995)
Beauveria pseudobassiana			
Isaria farinosa			
Isaria fumosorosea			
Beauveria brongniartii	N/A	Environmental	Munteanu et al. (2014)
Aspergillus parasiticus			
Penicillium steckii			
Scopulariopsis brevicaulis	nymphs, adults (females)	Tick colony	Bonnet et al. (2021)
**Nematoda**			

Cercopithifilaria rugosicauda	adults	Environmental	Winkhardt (1980)
**Arthropoda (Hymenoptera)**			
Ixodiphagus hookeri	nymphs	Environmental	Tijsse-Klasen et al. (2011)

Supplementary Table 3. Eukaryotic organisms detected in *Ixodes ricinus*.

References

Adamska, M., and Skotarczak, B. (2017). Molecular evidence for *Toxoplasma gondii* in feeding and questing *Ixodes ricinus* ticks. *Ticks Tick Borne Dis*. 8 (2), 259–261. doi: 10.1016/j.ttbdis.2016.11.009

Azagi, T., Jaarsma, R. I., Docters van Leeuwen, A., Fonville, M., Maas, M., Franssen, F. F. J., et al. (2021). Circulation of *Babesia* species and their exposure to humans through *Ixodes ricinus*. *Pathogens* 10 (4), 386. doi: 10.3390/pathogens10040386

Bonnet, S. I., Blisnick, T., Al Khoury, C., and Guillot, J. (2021). Of fungi and ticks: Morphological and molecular characterization of fungal contaminants of a laboratory-reared *Ixodes ricinus* colony. *Ticks Tick Borne Dis*. 12 (5), 101732. doi: 10.1016/j.ttbdis.2021.101732

Checa, R., López-Beceiro, A. M., Montoya, A., Barrera, J. P., Ortega, N., Gálvez, R., et al. (2018). *Babesia microti*-like piroplasm (syn. *Babesia vulpes*) infection in red foxes (*Vulpes vulpes*) in NW Spain (Galicia) and its relationship with *Ixodes hexagonus*. *Vet. Parasitol.* 252, 22–28. doi: 10.1016/j.vetpar.2018.01.011

Fernández, N., Revuelta, B., Aguilar, I., Soares, J. F., Zintl, A., Gray, J., et al. (2022). *Babesia* and *Theileria* identification in adult ixodid ticks from Tapada nature reserve, Portugal. *Pathogens* 11 (2), 222. doi: 10.3390/pathogens11020222

Hamšíková, Z., Kazimírová, M., Haruštiaková, D., Mahríková, L., Slovák, M., Berthová, L., et al. (2016a). *Babesia* spp. in ticks and wildlife in different habitat types of Slovakia. *Parasit. Vectors* 9 (1), 292. doi: 10.1186/s13071-016-1560-z

Hamšíková, Z., Silaghi, C., Rudolf, I., Vencľíková, K., Mahríková, L., Slovák, M., et al. (2016b). Molecular detection and phylogenetic analysis of *Hepatozoon* spp. in questing *Ixodes ricinus* ticks and rodents from Slovakia and Czech Republic. *Parasitol. Res*. 115 (10), 3897–3904. doi: 10.1007/s00436-016-5156-5

Kalsbeek, V., Frandsen, F., and Steenberg, T. (1995). Entomopathogenic fungi associated with *Ixodes ricinus* ticks. *Exp. Appl. Acarol*. 19 (1), 45–51. doi: 10.1007/bf00051936

Karlsson, M. E., and Andersson, M. O. (2015). *Babesia* species in questing *Ixodes ricinus*, Sweden. *Ticks Tick Borne Dis*. 7 (1), 10–12. doi: 10.1016/j.ttbdis.2015.07.016

Liberska, J., Michalik, J., Pers-Kamczyc, E., Wierzbicka, A., Lane, R. S., Rączka, G., et al. (2021). Prevalence of *Babesia canis* DNA in *Ixodes ricinus* ticks collected in forest and urban ecosystems in west-central Poland. *Ticks Tick Borne Dis*. 12 (5), 101786. doi: 10.1016/j.ttbdis.2021.101786

Luu, L., Bown, K. J., Palomar, A. M., Kazimı́rová, M., and Bell-Sakyi, L. (2020). Isolation and partial characterisation of a novel *Trypanosoma* from the tick *Ixodes ricinus*. *Ticks Tick Borne Dis*. 11 (5), 101501. doi: 10.1016/j.ttbdis.2020.101501

Munteanu, N. V., Mitkovets, P. V., Mitina, G. V., Movila, A., Tokarev, Y. S., and Leclerque, A. (2014). Prevalence of *Beauveria pseudobassiana* among entomopathogenic fungi isolated from the hard tick, *Ixodes ricinus*. *Ticks Tick Borne Dis*. 5 (6), 641–648. doi: 10.1016/j.ttbdis.2014.04.015

Nebbak, A., Dahmana, H., Almeras, L., Raoult, D., Boulanger, N., Jaulhac, B., et al. (2019). Co-Infection of bacteria and protozoan parasites in *Ixodes ricinus* nymphs collected in the Alsace region, France. *Ticks Tick Borne Dis.* 10 (6), 101241. doi: 10.1016/j.ttbdis.2019.06.001

Tijsse-Klasen, E., Braks, M., Scholte, E. J., and Sprong, H. (2011). Parasites of vectors–*Ixodiphagus hookeri* and its *Wolbachia* symbionts in ticks in the Netherlands. *Parasit. Vectors* 4, 228. doi: 10.1186/1756-3305-4-228

Weiser, J., and Rehácek, J. (1975). *Nosema slovaca* sp. n.: a second microsporidian of the tick *Ixodes ricinus*. *J. Invertebr Pathol*. 26 (3), 411. doi: 10.1016/0022-2011(75)90244-x

Weiser, J., Rehácek, J., Zizka, Z., Ciampor, F., and Kocianová, E. (1999). *Nosema slovaca* Weiser et Rehacek 1975 and *Unikaryon ixodis* [Weiser 1957] comb. n. in ixodid ticks. *Acta Parasitol*. 44 (2), 99–107.

Wilhelmsson, P., Pawełczyk, O., Jaenson, T. G. T., Waldenström, J., Olsen, B., Forsberg, P., et al. (2021). Three *Babesia* species in *Ixodes ricinus* ticks from migratory birds in Sweden. *Parasit. Vectors* 14 (1), 183. doi: 10.1186/s13071-021-04684-8

Winkhardt, H. J. (1980). Untersuchungen uber den Entwicklungszyklus von *Dipetalonema rugosicauda* (syn. *Wehrdikmansia rugosicauda*) (Nematoda; Filarioidea). II. Die Entwicklung von *Dipetalonema rugosicauda* im Zwischenwirt *Ixodes ricinus* und Untersuchungen uber das Vorkommen der Mikrofilarien im Reh (*Capreolus capreolus*). *Tropenmedizin und Parasitologie* 31 (1), 21–30.
